# SIRT5‐Mediated Desuccinylation of RAB7A Protects Against Cadmium‐Induced Alzheimer's Disease‐Like Pathology by Restoring Autophagic Flux

**DOI:** 10.1002/advs.202402030

**Published:** 2024-06-05

**Authors:** Ping Deng, Tengfei Fan, Peng Gao, Yongchun Peng, Min Li, Jingdian Li, Mingke Qin, Rongrong Hao, Liting Wang, Min Li, Lei Zhang, Chunhai Chen, Mindi He, Yonghui Lu, Qinlong Ma, Yan Luo, Li Tian, Jia Xie, Mengyan Chen, Shangcheng Xu, Zhou Zhou, Zhengping Yu, Huifeng Pi

**Affiliations:** ^1^ Department of Occupational Health (Key Laboratory of Electromagnetic Radiation Protection, Ministry of Education) Army Medical University (Third Military Medical University) Chongqing 400038 China; ^2^ Department of Oral and Maxillofacial Surgery The Second Xiangya Hospital of Central South University Changsha Hunan 410007 China; ^3^ Biomedical Analysis Center Army Medical University Chongqing 400038 China; ^4^ Basic Medical Laboratory General Hospital of Central Theater Command Wuhan 430070 China; ^5^ Hubei Key Laboratory of Central Nervous System Tumour and Intervention Wuhan 430070 China; ^6^ Center of Laboratory Medicine Chongqing Prevention and Treatment Center for Occupational Diseases Chongqing Key Laboratory of Prevention and Treatment for Occupational Diseases and Poisoning Chongqing 400060 China; ^7^ Center for Neuro Intelligence School of Medicine Chongqing University Chongqing 400030 China; ^8^ State Key Laboratory of Trauma and Chemical Poisoning Army Medical University Chongqing 400038 China

**Keywords:** alzheimer's disease, autophagy, cadmium, rab7a, sirt5

## Abstract

Cadmium (Cd) is a neurotoxic contaminant that induces cognitive decline similar to that observed in Alzheimer's disease (AD). Autophagic flux dysfunction is attributed to the pathogenesis of AD, and this study aimed to investigate the effect of autophagy on environmental Cd‐induced AD progression and the underlying mechanism. Here, Cd exposure inhibited autophagosome‐lysosome fusion and impaired lysosomal function, leading to defects in autophagic clearance and then to APP accumulation and nerve cell death. Proteomic analysis coupled with Ingenuity Pathway Analysis (IPA) identified SIRT5 as an essential molecular target in Cd‐impaired autophagic flux. Mechanistically, Cd exposure hampered the expression of SIRT5, thus increasing the succinylation of RAB7A at lysine 31 and inhibiting RAB7A activity, which contributed to autophagic flux blockade. Importantly, SIRT5 overexpression led to the restoration of autophagic flux blockade, the alleviation of Aβ deposition and memory deficits, and the desuccinylation of RAB7A in Cd‐exposed FAD^4T^ mice. Additionally, SIRT5 levels decrease mainly in neurons but not in other cell clusters in the brains of AD patients according to single‐nucleus RNA sequencing data from the public dataset GSE188545. This study reveals that SIRT5‐catalysed RAB7A desuccinylation is an essential adaptive mechanism for the amelioration of Cd‐induced autophagic flux blockade and AD‐like pathogenesis.

## Graphical abstract

The underlying mechanism of autophagy on environmental cadmium‐induced Alzheimer's disease progression is vague. The findings indicated that SIRT5 desuccinylated and activated RAB7A at the K31 residue to rescue the cadmium‐provoked Alzheimer's disease progression by restoring the autophagic flux, and identified potential targets for the treatment of environmental contaminant‐associated brain degeneration. BioRender is used to draw the graphical abstract.

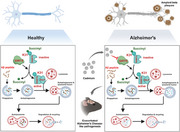

## 1 Introduction

Cadmium (Cd) is a naturally occurring, water‐soluble heavy metal.^[^ ^1^ ^]^ In various natural and human‐made processes (i.e., fossil fuel combustion, mining, the manufacture of batteries and pigments, and plant growth), Cd is released into the atmosphere as an environmental pollutant.^[^ ^2^ ^]^ Cd can cross the blood‐brain barrier, eventually accumulating in the brain and causing neurotoxicity.^[^ ^3^ ^]^ Cd exposure is a potential environmental risk factor that contributes to the progression of Alzheimer's disease (AD), a devastating neurodegenerative disorder.^[^ ^4^ ^]^ Several studies have revealed that the blood Cd levels of AD patients are clearly greater than those of healthy controls,^[^ ^5^ ^]^ and AD mortality is significantly associated with the blood Cd levels among older adults in the US.^[^ ^6^ ^]^ In animal studies, Cd exposure has been linked to increased amyloid beta peptides (AβPs) and β‐amyloid (Aβ) deposition which is one of the principal pathological hallmarks of AD.^[^ ^7^ ^]^ Cd‐exposed amyloid precursor protein (APP)/presenilin 1 (PS1) transgenic mice resulted in notable impairments in cognitive function, increased levels of Aβ_1‐42_, decreased levels of α‐secretase protein and soluble AβPPα (sAβPPα), and ultimately an escalation in Aβ plaque deposition.^[^ ^8^ ^]^ Notarachille et al. reported that Cd interacted with the AβPs, reducing channel activity and forming large amorphous aggregates in solution that are prone to precipitate, subsequently promoting the formation of Aβ plaques.^[^ ^9^ ^]^ In addition, Cd treatment in the media strongly elevated Aβ‐like immunoreactivity in mouse organotypic brain slices.^[^ ^10^ ^]^ Discovering the underlying pathogenesis of AD linked to Cd would expand our understanding of its pathogenesis and allow the discovery of new treatment targets and strategies for environmentally relevant toxic metal‐linked AD.

Autophagy is an evolutionarily conserved process in which abnormal proteins or defective organelles are degraded in lysosomes and basic components are rejuvenated in eukaryotic cells.^[^ ^11^ ^]^ The accumulated body of evidence shows that impaired autophagy contributes to the pathogenesis of AD, as evidenced by the accumulation of immature autophagic vacuoles (AVs) in dystrophic neurites^[^ ^12^ ^]^ and even prior to the occurrence of synaptic and neuronal degeneration in AD mice.^[^ ^13^ ^]^ In addition, growing studies reveal that autophagy is involved in Aβ metabolism, potentially through the regulation of both its production and elimination.^[^ ^14^ ^]^ Di Meco et al. revealed the presence of APP and the 4 subunits of the γ‐secretase complex within autophagosomes, indicating that the autophagic pathway may contribute to the production of Aβ peptides.^[^ ^15^ ^]^ Autophagy elevates the degradation and elimination of amyloid‐β precursor protein (APP),^[^ ^16^ ^]^ along with its various cleavage products, such as Aβ^[^ ^17^ ^]^ and APP‐cleaved C‐terminal fragment (APP‐CTF).^[^ ^18^ ^]^ In addition, previous studies have demonstrated that the administration of rapamycin, a specific agonist of autophagy, to AD mice results in a significant reduction in intracellular Aβ and extracellular amyloid deposition in the brain and improved cognitive function.^[^ ^19^ ^]^ These findings suggest that impaired autophagy might be responsible for the accumulation of APP and Aβ and that autophagy activation could be a potential treatment for AD.

Sirtuin 5 (SIRT5) is a member of the sirtuin family of nicotinamide adenine dinucleotide (NAD^+^)‐dependent protein deacetylases, which plays a role in regulating the physicochemical properties of proteins through posttranslational modifications (PTMs).^[^ ^20^ ^]^ SIRT5 has been shown to modulate various biological processes through its weak deacetylase activity but strong desuccinylase, demalonylase, and deglutarylase activities.^[^ ^21^ ^]^ The site H158 in SIRT5 is characterized by high conservation and functional significance, with the mutant SIRT5‐H158Y exhibiting a loss of lysine deacylation activity.^[^ ^22^ ^]^ Additionally, SIRT5 has been implicated in the pathogenesis of several human diseases, including AD, Parkinson's disease (PD), and cancer.^[^ ^23^ ^]^ Wu et al. observed a clear downregulation of SIRT5 in the brains of mice with AD and found that ectopic expression of SIRT5 counteracts the brain injuries induced by oxidative stress while also suppressing the activation of microglia and astrocytes.^[^ ^24^ ^]^ In addition, the administration of exogenous Aβ1‐42 oligomers (AβO) significantly decreased the expression of SIRT5 in neuronal SH‐SY5Y cells.^[^ ^25^ ^]^ Furthermore, SIRT5 could disrupt autophagy membrane formation, blocking the autophagic flux and inhibiting the growth of breast tumor cells that rely on deacetylation activities.^[^ ^26^ ^]^ The inhibition of SIRT5 might impede the desuccinylation of glutaminase (GLS) at the Lys158 and Lys164 sites, leading to the stabilization of GLS and the promotion of glutaminolysis, ultimately enhancing autophagy.^[^ ^27^ ^]^ Huang et al. demonstrated that SIRT5 had the potential to mitigate morphine tolerance through the modulation of hydroxyl‐CoA dehydrogenase alpha subunit (HADHA) desuccinylation, consequently augmenting autophagic flux in the rat brain.^[^ ^28^ ^]^ Although a previous study indicated that SIRT5 overexpression can improve the progression of AD by promoting autophagy,^[^ ^24^ ^]^ the precise role and underlying mechanism of SIRT5 in autophagy during Cd exposure‐induced AD progression remain largely vague.

Lysosomal Ras‐associated protein 7a (RAB7A) belongs to the small GTPase family, the members of which are thought to function as molecular switches regulating vesicular membrane traffic by shifting between their GDP‐bound (inactive) and GTP‐bound (active) states.^[^ ^29^ ^]^ Growing evidence implies that RAB7A controls vesicle formation, transport, and fusion processes and thereby plays a crucial role in the regulation of autophagosome‐lysosome fusion and autolysosomal maturation.^[^ ^30^ ^]^ RAB7A facilitates the recruitment of Pleckstrin homology domain‐containing protein family member 1 (PLEKHM1) to interact with the tethering factor, homotypic fusion, and protein sorting (HOPS) complex, to lysosome membranes to control the autophagosome‐lysosome fusion.^[^ ^31^ ^]^ Another critical RAB7A effector protein, RAB7A‐interacting lysosomal protein (RILP), is apparently recruited to active RAB7A and forms a complex that mediates lysosomal transport by facilitating the recruitment of dynein‐dynactin motors.^[^ ^32^ ^]^ Additionally, RAB7A is recognized as an essential regulator of lysosomal biogenesis and function.^[^ ^33^ ^]^ Bucci et al. reported that RAB7A regulated the aggregation and fusion processes of late endocytic structures/lysosomes, playing a crucial role in maintaining the perinuclear lysosome compartment and preserving lysosomal acidity.^[^ ^34^ ^]^ Hu et al. demonstrated that reducing RAB7A led to lower levels of cathepsin B (CTSB) and cathepsin D (CTSD) proteins, which are crucial lysosomal proteases that participate in lysosomal degradation while increasing RAB7A reversed the decreased levels of CTSB and CTSD.^[^ ^35^ ^]^ RAB7A activity is also regulated by numerous PTMs, such as geranylgeranylation^[^ ^36^ ^]^ and phosphorylation.^[^ ^37^ ^]^ Moreover, a growing body of research has shown that the process of RAB7A‐mediated autophagy is intricately linked to numerous pathological mechanisms observed in several neurological disorders, including Huntington's disease, PD, and AD.^[^ ^38^ ^]^ In brief, these reports suggest that RAB7A, which functions as a regulator of neuronal signaling, holds promise for identifying innovative approaches for treating autophagy‐associated neurological disorders.

In the present study, it was observed that exposure to Cd significantly inhibited the expression of SIRT5, thereby impeding autophagic flux and causing the accumulation of APP and neurotoxicity through impairment of autophagosome‐lysosome fusion and lysosomal function. Quantitative succinylome analysis revealed that reduced SIRT5 expression resulted in elevated succinylation of RAB7A at lysine 31 (K31), which reduced the activity of RAB7A and hindered its recruitment to RILP, leading to the blockade of autophagic flux and ultimately contributed to the progression of AD pathology and cognitive decline induced by Cd exposure. Ultimately, we substantiated the effectiveness of interventions aimed at enhancing SIRT5 expression in the treatment of Cd‐exacerbated disease progression in FAD^4T^ mice and thus identified a novel therapeutic approach for AD linked to environmentally relevant toxic metals.

## 2 Results

### 2.1 Cd Exposure Promotes APP Accumulation and Autophagic Flux Blockage in Neuro‐2a Cells

**Figure 1:**
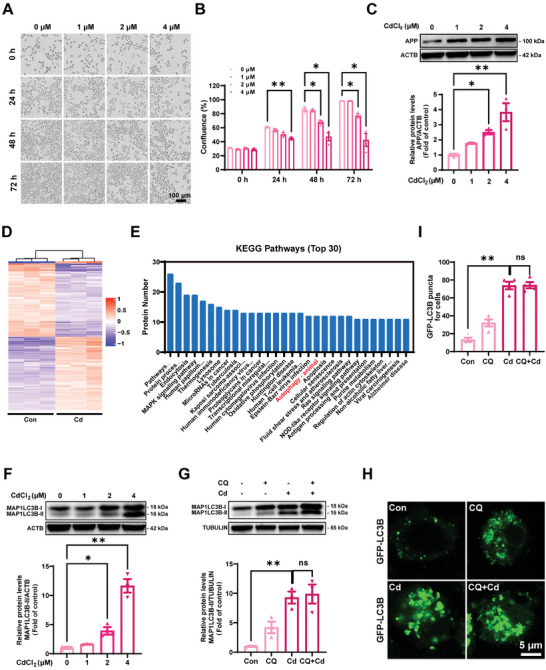
Cd exposure increases APP expression and blocks autophagic flux in Neuro‐2a cells A, B) The confluence (% of the surface area of cells) of Neuro‐2a cells treated with CdCl_2_ at different concentrations (0, 1, 2, and 4 µM) for 0, 24, 48, or 72 h was detected with an Incucyte ZOOM System. Scale bar: 100 µm. C) Representative immunoblot and quantification analysis of APP in Neuro‐2a cells treated with CdCl_2_ at different concentrations (0, 1, 2, and 4 µM). D) Heatmap of differentially expressed proteins in Neuro‐2a cells treated with or without CdCl_2_ (4 µM, 72 h). E) Top 30 pathways identified by KEGG enrichment analysis. (F) Representative immunoblot and quantification analysis of MAP1LC3B in Neuro‐2a cells treated with Cd at different concentrations (0, 1, 2, and 4 µM) for 72 h. Representative immunoblot and quantification analysis of MAP1LC3B G) and representative images and quantification of GFP‐LC3B puncta (H, I) in Neuro‐2a cells treated with or without CdCl_2_ (4 µM, 72 h) in the absence or presence of CQ (25 µM, 72 h). Scale bar: 5 µm. ^*^ *p *< 0.05 and ^**^ *p *< 0.01 versus the control group. ns: not significant.

The neurotoxicity of Cd was assessed with an IncuCyte ZOOM Live Cell Analysis System, which revealed a clear decrease in Neuro‐2a cell viability in a dose‐ and time‐dependent manner (0, 1, 2, and 4 µM cadmium chloride (CdCl_2_) for 24, 48 and 72 h), respectively) (**Figure** **1**A,B). Additionally, a dose‐dependent increase in APP expression was observed in Neuro‐2a cells exposed to Cd for 72 h (Figure 1C). Kyoto Encyclopedia of Genes and Genomes (KEGG) pathway analysis of the proteomic profiles confirmed that proteins involved in “autophagy” were obviously enriched in the Cd‐treated Neuro‐2a cells compared with the control cells (Figure 1D,E). Moreover, Cd provoked autophagosome accumulation in a dose‐dependent manner, as evidenced by dose‐dependent changes in the MAP1LC3B‐II levels (Figure 1F). Chloroquine (CQ) effectively hinders the fusion process between autophagosomes and lysosomes, thereby suppressing autophagy during its advanced stage.^[^ ^39^ ^]^ Our study demonstrated that the addition of 25 or 37.5 µM CQ combined with Cd did not have any impact on the MAP1LC3B‐II levels or the number of puncta, suggesting that Cd impedes the degradation of autophagic contents (Figure 1G—I; Figure S1, Supporting Information).

Given that autophagy is a complex process consisting of multiple stages, our study aimed to determine which specific stage is impaired by Cd to result in the blockade of autophagic flux. We first transfected small interfering RNA (siRNA) targeting autophagy‐related 5 (*Atg5*) into Neuro‐2a cells with or without Cd exposure. Knockdown of *Atg5* resulted in a reduction in the accumulation of MAP1LC3B‐II triggered by Cd, suggesting that Cd does not affect phagophore formation (Figure S2A, Supporting Information). Additionally, treatment with the autophagy inhibitor 3‐MA yielded similar findings (Figure S2B, Supporting Information). Cargo incorporation occurs during autophagosome maturation, which was evaluated by the colocalization of GFP‐LC3B and sequestosome 1 (SQSTM1).^[^ ^40^ ^]^ Cd exposure also resulted in greater colocalization of GFP‐LC3B with SQSTM1 than that observed in the control cells (Figure S2C,D, Supporting Information). Increased levels of lysosome‐associated membrane protein 1 (LAMP1), a crucial protein involved in lysosome formation, were observed in Cd‐exposed Neuro‐2a cells compared with control cells (Figure S3A, Supporting Information). A decrease in the colocalization of the autophagosome marker GFP‐LC3B with the lysosomal marker lysosomal‐associated membrane protein 2 (LAMP2) indicated an apparent reduction in the fusion of autophagosomes and lysosomes in Cd‐treated Neuro‐2a cells compared with control cells (Figure S3B–D, Supporting Information). The acidotropic probe LysoSensor Green DND‐189 dye has been observed to accumulate in acidic organelles, specifically lysosomes, and to exhibit an increase in fluorescence intensity corresponding to acidification.^[^ ^40^ ^]^ Cd exposure resulted in a dose‐dependent reduction in LysoSensor Green DND‐189 fluorescence, indicating a perturbation in the lysosomal pH in Neuro‐2a cells compared with control cells (Figure S3E, Supporting Information). Furthermore, Cd treatment induced an increase in the ratio of yellow to red puncta in Neuro‐2a cells compared with control cells, as observed through the utilization of the RFP‐GFP‐LC3B reporter system, and this increase indicated a noticeable decrease in the acidity of autolysosomes (Figure S3F,G, Supporting Information). Additionally, the assessment of CTSB and CTSD activity further supported the notion of impaired lysosomal degradation in Cd‐treated Neuro‐2a cells (Figure S3H,I, Supporting Information). These findings suggested that Cd triggered autophagic flux blockade by obstructing autophagosome‐lysosome fusion and compromising lysosomal function in Neuro‐2a cells. Collectively, these results offer compelling evidence indicating that Cd exposure exacerbates APP accumulation and blocks autophagic flux in Neuro‐2a cells.

### 2.2 SIRT5‐Dependent Deacylation Deficiency Participates in Cd‐Evoked APP Accumulation and Autophagic Flux Blockage

**Figure 2:**
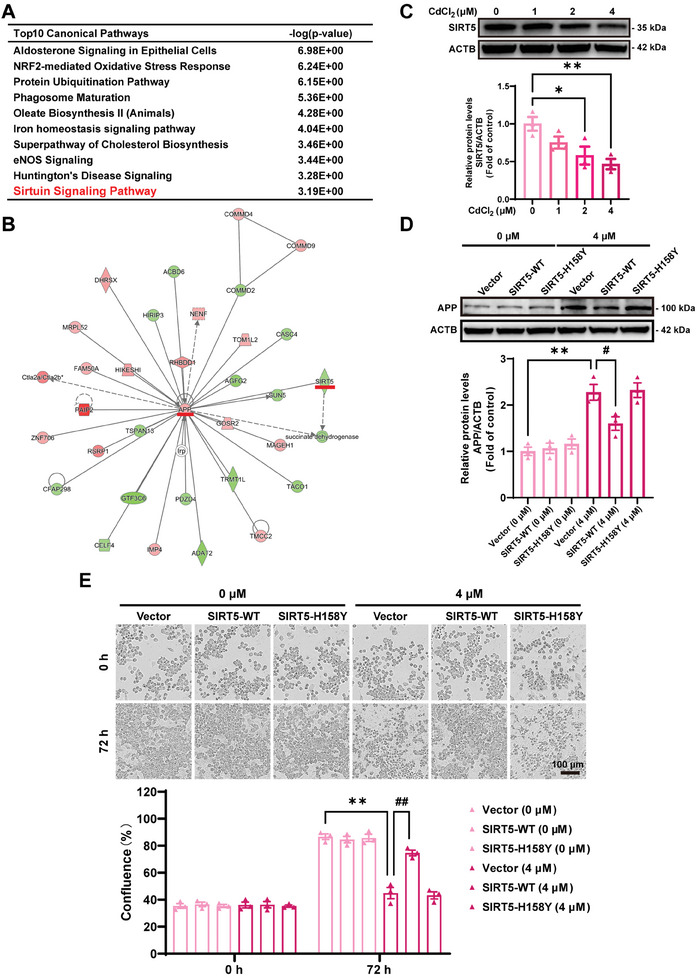
SIRT5‐dependent deacylation plays an important role in Cd‐induced APP accumulation and nerve cell death. A) Top 10 canonical pathways identified by IPA. B) Predicted crucial network identified by IPA. C) Representative immunoblot and quantification of SIRT5 in neuro‐2a cells treated with Cd at different concentrations (0, 1, 2, and 4 µM) for 72 h. D, E Neuro‐2a cells were transfected with the control vector (Vector), wild‐type SIRT5‐overexpression plasmid (SIRT5‐WT) or inactive SIRT5 mutant plasmid (SIRT5‐H158Y) and treated with or without CdCl_2_ (4 µM) for 72 h. (D) Representative immunoblot and quantification of APP in Neuro‐2a cells. (E) Representative images and quantification of Neuro‐2a cell confluence. Scale bar: 100 µm. ^*^ *p *< 0.05 and ^**^ *p* < 0.01 versus the control groups; ^#^ *p* <0.05 and ^##^ *p* <0.01 versus the Cd‐exposed groups.

**Figure 3:**
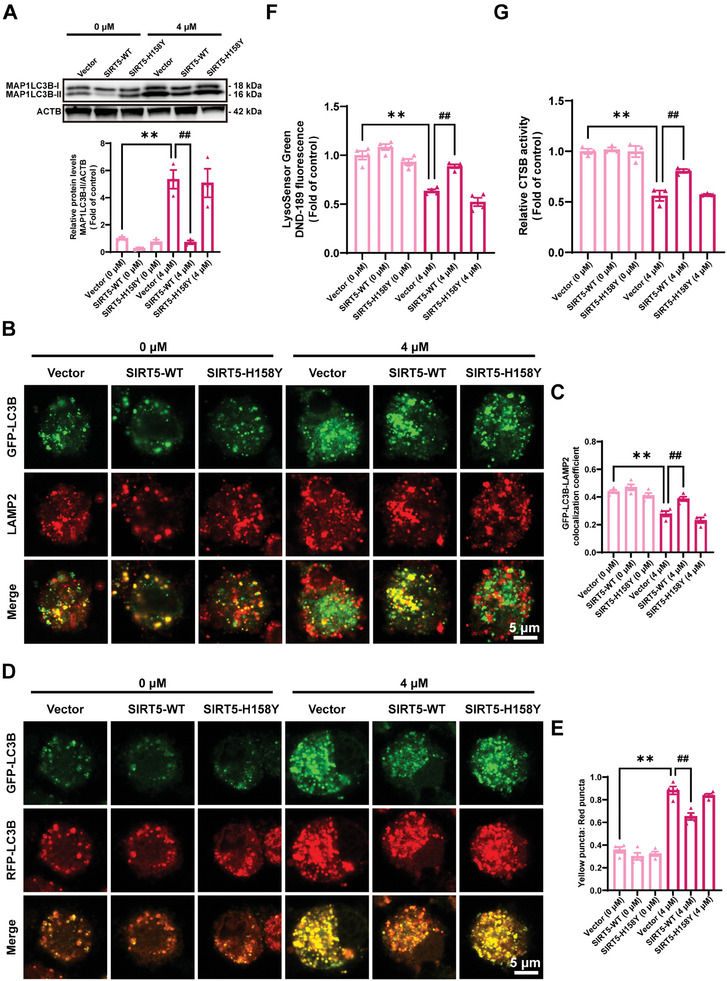
SIRT5 but not the inactive mutant SIRT5‐H158Y overexpression antagonizes Cd‐induced autophagic flux blockade. In A‐G, all Neuro‐2a cells were transfected with Vector, SIRT5‐WT, or the SIRT5‐H158Y plasmid and treated with or without CdCl_2_ (4 µM) for 72 h. A) Representative immunoblot and quantification of MAP1LC3B. B) Representative immunofluorescence images of Neuro‐2a cells transfected with the GFP‐LC3B plasmid and detected with an anti‐LAMP2 antibody. Scale bar: 5 µm. C) Statistical analysis of the colocalization coefficient of GFP‐LC3B puncta and LAMP2 puncta. D, E) Representative immunofluorescence and statistical analysis (yellow puncta: red puncta) of Neuro‐2a cells transfected with the tandem sensor RFP‐GFP‐LC3B for 24 h. Scale bar: 5 µm. F) LysoSensor DND‐189 fluorescence intensity and CTSB activity G) in Neuro‐2a cells. ^**^ *p *< 0.01 versus the control groups; ^##^ *p* < 0.01 versus the Cd‐exposed groups.

To probe the potential molecular mechanism underlying Cd‐induced neurotoxicity, differentially expressed proteins obtained by TMT‐based quantitative proteomic analysis were subjected to Ingenuity Pathway Analysis (IPA). Canonical pathway analysis revealed that the sirtuin signaling pathway was among the top 10 pathways (**Figure** **2**A). Subsequently, a significant correlation between the upregulation of APP and the reduction in the SIRT5 levels was identified by the molecular interaction network, suggesting that SIRT5 may play a vital role in APP accumulation (Figure 2B). We found that the protein level of SIRT5 dose‐dependently decreased in Cd‐treated Neuro‐2a cells (Figure 2C). SIRT5 modulates the properties and functions of proteins by strongly catalyzing lysine modification, desuccinylation, demalonylation, and deglutarylation.^[^ ^41^ ^]^ To explore whether enzymatic activity is required for the action of SIRT5 in autophagic metabolism and Cd‐induced neurotoxicity, Neuro‐2a cells treated with or without Cd were transfected with plasmids containing wild‐type SIRT5 (SIRT5‐WT), the catalytically inactive mutant SIRT5‐H158Y or empty pcDNA3.1 (Vector). The overexpression of SIRT5‐WT, but not SIRT5‐H158Y, counteracted the increase in APP accumulation and the decrease in cell viability induced by Cd treatment (Figure 2D,E). Furthermore, the overexpression of SIRT5‐WT, but not SIRT5‐H158Y, strongly reversed the increase in MAP1LC3B‐II levels and the decrease in colocalization between GFP‐LC3B and LAMP2 induced by Cd treatment (**Figure** **3**A–C). Moreover, SIRT5‐WT overexpression, but not SIRT5‐H158Y, improved lysosomal acidification and promoted lysosomal proteolytic activity (Figure 3D–G). These results confirmed that SIRT5 mitigated the neurotoxic effects of Cd through its deacylation activity to enhance autophagy.

### 2.3 RAB7A is Hypersuccinylated at the K31 Site in Cd‐Exposed Neuro‐2a Cells

**Figure 4:**
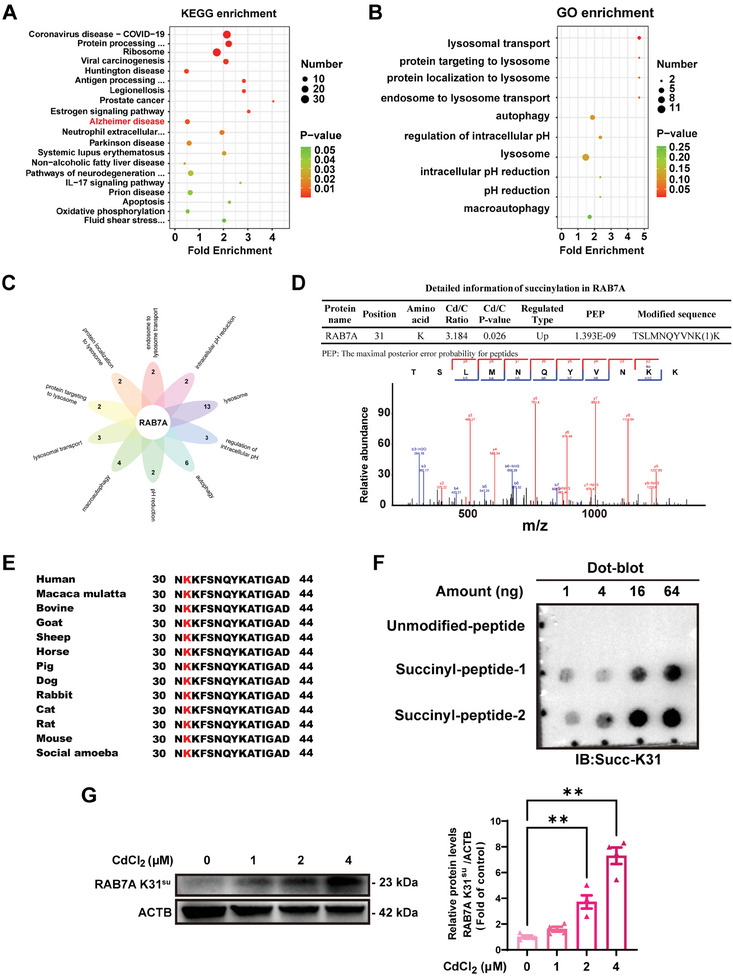
RAB7A is hypersuccinylated at the K31 site in Cd‐exposed Neuro‐2a cells. A) Top 20 pathways according to a KEGG enrichment analysis of the proteins with differentially expressed succinylated modification sites. B) Enriched GO terms linked to autophagy‐lysosome machinery obtained from proteins with differentially expressed succinylated modification sites. C) Overlap of the GO terms identified in the GO enrichment analysis. D) Results of a mass spectrometry analysis of succinylated RAB7A at the K31 site. E) K31 (marked in red) is evolutionarily conserved in RAB7A. F) The anti‐RAB7A‐succinyl‐K31 antibody was characterized through a dot blot assay. G) Representative immunoblot and quantification of RAB7A K31^su^ in Neuro‐2a cells treated with Cd at different concentrations (0, 1, 2, and 4 µM) for 72 h. ^**^ *p *< 0.01 versus the control groups.

Considering SIRT5 strongly induces desuccinylation, demalonylation or deglutarylation of acyl‐lysine residues, the levels of these PTMs were assessed in Neuro‐2a cells treated or not treated with Cd for 72 h. Compared with those in the control group, the succinylation levels of proteins exhibited a clear dose‐dependent increase in response to Cd treatment, whereas the levels of malonylation and glutarylation did not significantly change (Figure S4, Supporting Information). To identify the lysine‐succinylated (K‐Succ) sites in proteins that were significantly altered by Cd treatment, a 4D‐label‐free quantitative proteomic analysis of succinylation comparing Cd‐treated Neuro‐2a cells and control cells was conducted via LC‒MS/MS (Figure S5A–D, Supporting Information). The majority of the modification sites were found within the K^su^_XXXXXXX_K motif (Figure S5E,F, Supporting Information). Quantitative data with a *p*‐value <0.05 and a ratio >1.5 or <0.667 were considered to indicate differential succinylation; 559 sites in 233 proteins exhibited increased succinylation, and 25 sites in 19 proteins exhibited decreased succinylation (Figure S5G–I). KEGG enrichment analysis was conducted to further assess the altered pathways involving these hypersuccinylated proteins, and the results indicated that the AD pathway was among the top 20 enriched terms (**Figure** **4**A). The Gene Ontology (GO) terms involved in autophagy‐lysosome machinery were chosen for further investigation (Figure 4B). RAB7A was the only gene that overlapped with all selected GO terms and thus played an essential role in Cd‐induced autophagic flux blockade (Figure 4C). Lysine succinylome analysis revealed a single lysine site, Lys31 (K31), in RAB7A, which was markedly upregulated by Cd treatment in Neuro‐2a cells (Figure 4D). Lys31 is highly conserved in RAB7A orthologues from humans to social amoebas, indicating that it is essential for the function of RAB7A (Figure 4E). To validate the occurrence of succinylation at the Lys31 site in RAB7A, we developed an antibody that specifically identified succinylated K31 in RAB7A (Figures S6 and S7, Tables S1–S3, Supporting Information). Dot blot detection demonstrated that the anti‐Succ‐K31‐specific antibody selectively recognized the K31‐succinylated peptide (1 and 2) but did not react with the unsuccinylated control peptide (Figure 4F), suggesting the high specificity of the developed antibody. Western blotting utilizing this site‐specific antibody revealed that the RAB7A K31^su^ level dose‐dependently increased in Neuro‐2a cells treated with Cd (Figure 4G). Collectively, these observations reveal that Lys31 is a critical succinylation site in RAB7A that may be involved in Cd‐induced autophagic flux blockade and neurotoxicity.

### 2.4 Desuccinylation of RAB7A at the K31 Site Counteracts the Cd‐Induced Autophagic Flux Blockage and Neurotoxicity

**Figure 5:**
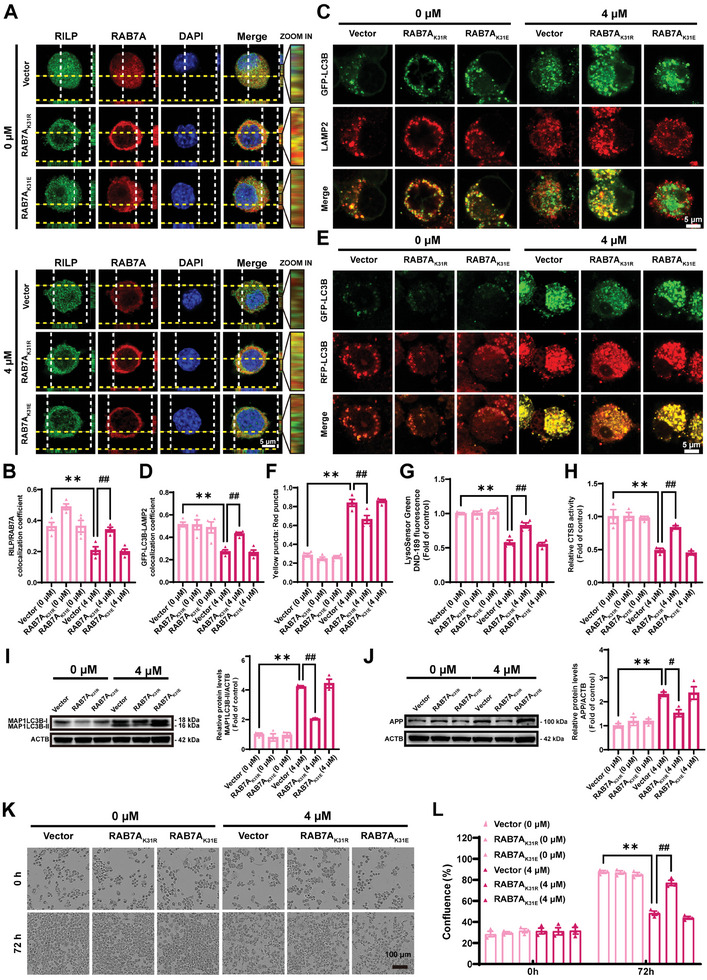
Desuccinylation of RAB7A at the K31 site counteracts Cd‐induced autophagic flux blockade and neurotoxicity. All Neuro‐2a cells were transfected with the control vector, RAB7A_K31R_ (mimicking the desuccinylated state) or RAB7A_K31E_ (mimicking the succinylated state) plasmids and treated with or without CdCl_2_ (4 µM) for 72 h. A, B) Representative immunofluorescence images and statistical results of the colocalization of RILP and RAB7A. Scale bar: 5 µm. C, D) Representative immunofluorescence images and statistical analysis of the colocalization coefficients of GFP‐LC3B puncta and LAMP2 puncta. Scale bar: 5 µm. E, F) Representative immunofluorescence images and the ratio of yellow puncta to red puncta indicated by Tandem Sensor RFP‐GFP‐LC3B. Scale bar: 5 µm. LysoSensor DND‐189 fluorescence intensity G) and CTSB activity H) detection. Representative immunoblot and quantification of MAP1LC3B I) and APP (J) in Neuro‐2a cells. K, L) Alterations in the Neuro‐2a cell confluence and statistical analysis. Scale bar: 100 µm. ^**^ *p* < 0.01 versus the control groups; ^#^ *p *< 0.05 and ^##^ *p* < 0.01 versus the Cd‐exposed groups.

To verify the role of the desuccinylation of RAB7A K31 in Cd‐induced autophagic flux blockade and neurotoxicity, 2 mutant plasmids, RAB7A_K31R_ [lysine (K) 31 was substituted with arginine (R); mimicking the desuccinylated state)] and RAB7A_K31E_ [lysine (K) 31 was substituted with glutamic acid (E); mimicking the negatively charged succinylation modification)], were constructed and transfected into Neuro‐2a cells treated with or without Cd, respectively. The activation of RAB7A facilitates the binding of its crucial effector, RILP, thereby enabling the evaluation of the Rab GTPase activation status through RILP binding.^[^ ^42^ ^]^ Compared with empty pcDNA3.1 (Vector), RAB7A_K31R_ markedly reversed the Cd‐induced decrease in the colocalization coefficient between RILP and RAB7A, whereas the presence of RAB7A_K31E_ did not exert any discernible effect, indicating that desuccinylation of RAB7A K31 facilitated its activity (**Figure** **5**A,B). Notably, RAB7A_K31R_, which mimics the desuccinylated state, but not RAB7A_K31E_, which mimics the succinylated state, markedly reversed the decrease in fusion between autophagosomes and lysosomes induced by Cd, as indicated by an increase in the colocalization coefficient between GFP‐LC3B and LAMP2 (Figure 5C,D). Similarly, RAB7A_K31R_ overexpression clearly facilitated more red puncta versus yellow puncta and an increase in the fluorescence intensity was related to lower PH in acidic organelles, such as lysosomes, and the proteolytic activity of CTSB in Cd‐treated Neuro‐2a cells (Figure 5E–H). However, little alteration was observed in the overexpression of RAB7A_K31E_ in Cd‐treated Neuro‐2a cells, as compared to the control vector (Figure 5E–H). Western blotting exhibited a coincident alteration in the MAP1LC3B‐II levels in Neuro‐2a cells transfected with RAB7A_K31R_, RAB7A_K31E_, or the control vector in the absence or presence of Cd (Figure 5I). These results suggested that the autophagic machinery was restored by RAB7A_K31R_ in response to Cd exposure. Interestingly, these alterations triggered by RAB7A_K31R_, but not RAB7A_K31E_, abrogated the abnormal accumulation of APP and cytotoxicity induced by Cd in Neuro‐2a cells (Figure 5J–L). Collectively, these data demonstrated that RAB7A with desuccinylation at the K31 site can abrogate the Cd‐induced autophagic flux blockade and neurotoxicity.

### 2.5 SIRT5 Desuccinylates and Reinforces RAB7A Activities in Cd‐Exposed Neuro‐2a Cells

**Figure 6:**
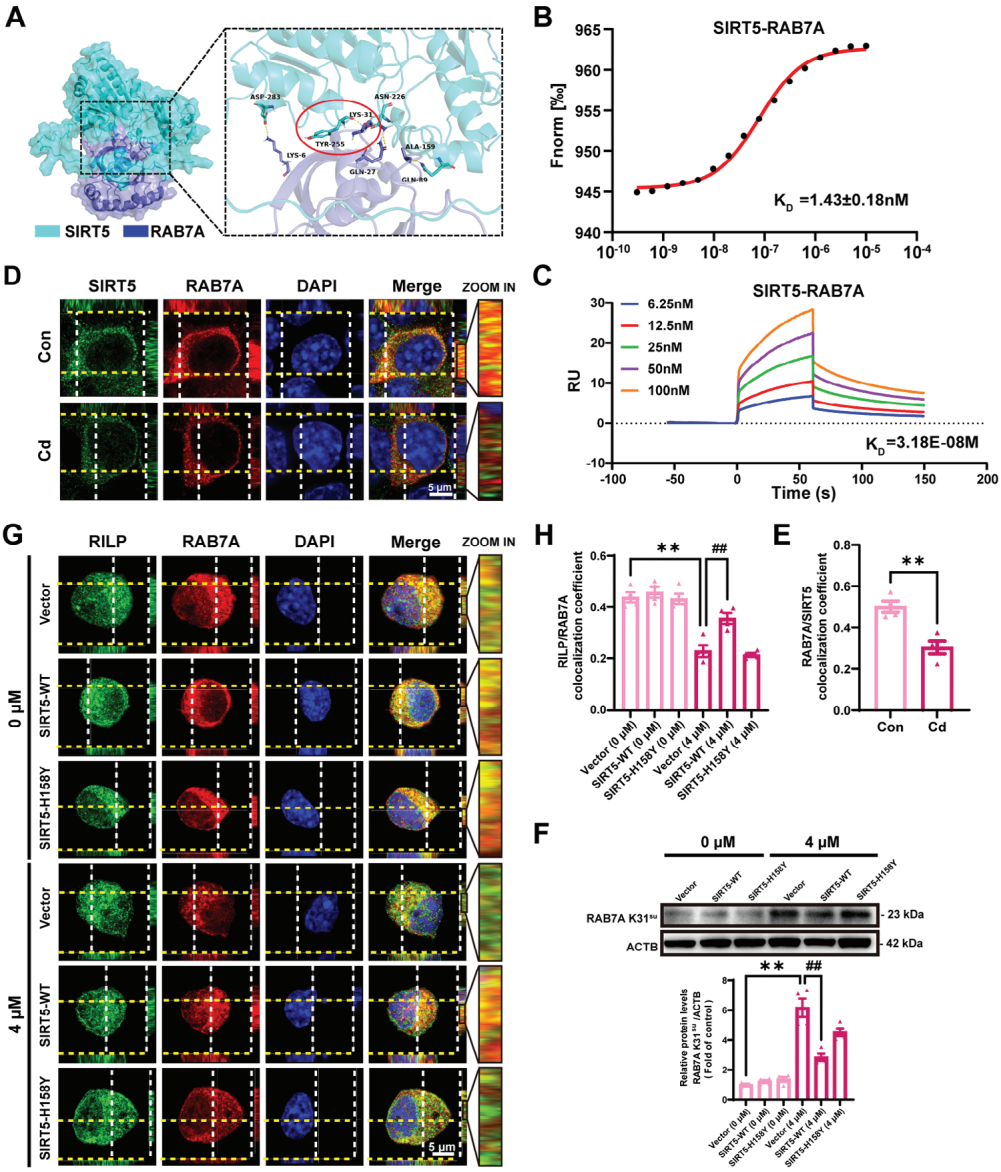
SIRT5 interacts with and desuccinylates RAB7A at the Lys31 site and antagonizes Cd‐inhibited RAB7A activity. A) Molecular docking model of RAB7A binding to the SIRT5 domain. The yellow dashed lines indicate hydrogen bonds. B) MST analysis of the direct interaction between SIRT5 and RAB7A. C) SPR analysis of the binding affinity curve of SIRT5 for RAB7A. D, E) Immunofluorescence analysis of endogenous SIRT5 and RAB7A in Neuro‐2a cells treated with or without CdCl_2_ (4 µM) for 72 h. Scale bar: 5 µm. F‐H Neuro‐2a cells were transfected with the control vector or the SIRT5‐WT or SIRT5‐H158Y plasmid and treated with or without CdCl_2_ (4 µM) for 72 h. F) Representative immunoblot and quantification of RAB7A K31^su^ in Neuro‐2a cells. G, H) Immunofluorescence analysis of endogenous RILP and RAB7A in Neuro‐2a cells. Scale bar: 5 µm. ^**^ *p* < 0.01 versus the control groups; ^##^ *p* < 0.01 versus the Cd‐exposed groups.

To ascertain whether SIRT5 desuccinylates the K31 site in RAB7A and regulates RAB7A function, an initial investigation was conducted to determine the direct interaction between SIRT5 and RAB7A. First, we performed molecular docking analysis using the crystal structure of SIRT5 predicted by the AlphaFold protein structure database and the crystal structure of RAB7A obtained from the PubChem protein database (PDB ID: 5JRH). Multiple groups of residues were identified as being involved in the formation of hydrogen bonds between RAB7A and SIRT5 (**Figure** **6**A; Table S4, Supporting Information). Notably, a favorable hydrogen bond was observed between Lys31 of RAB7A and Tyr255 of SIRT5 (Figure 6A; Table S4, Supporting Information). Subsequently, microscale thermophoresis (MST) was employed using purified recombinant protein, revealing the strong binding affinity of SIRT5 for RAB7A, with an equilibrium dissociation constant (*K*_D_) of ≈1.43 nM (Figure 6B; Figure S8, Supporting Information). Then, a surface plasmon resonance (SPR) assay confirmed that SIRT5 is strongly bound to RAB7A (Figure 6C, Figure S7, Supporting Information). Furthermore, immunofluorescence assays uncovered an obvious colocalization of intracellular SIRT5 with RAB7A, and this colocalization was markedly decreased in Cd‐treated Neuro‐2a cells compared with control cells (Figure 6D,E). To explore the modulatory effect of SIRT5 on the succinylation of RAB7A at K31, we utilized an anti‐RAB7A Succ‐K31‐specific antibody to assess the levels of RAB7A K31^su^ following transfection with SIRT5‐WT, SIRT5‐H158Y, or a control vector in Neuro‐2a cells in the absence or presence of Cd. Indeed, SIRT5‐WT overexpression abolished the Cd‐induced increase in the levels of RAB7A K31^su^ but not the inactive SIRT5‐H158Y mutant, suggesting that K31 succinylation is a physiological PTM of RAB7A and is directly regulated by SIRT5‐dependent desuccinylation (Figure 6F). Moreover, the overexpression of SIRT5‐WT, but not SIRT5‐H158Y, induced more RAB7A binding to RILP, which mitigated the disturbance in colocalization between RAB7A and RILP triggered by Cd (Figure 6G,H). Overall, these results revealed that SIRT5 desuccinylated the K31 site of RAB7A and thus regulates RAB7A activity in Cd‐treated Neuro‐2a cells.

### 2.6 SIRT5 Overexpression Protects against Cd‐Induced AD‐like Pathology, Cognitive Decline, and Autophagic Flux Blockage in FAD^4T^ Transgenic Mice

**Figure 7:**
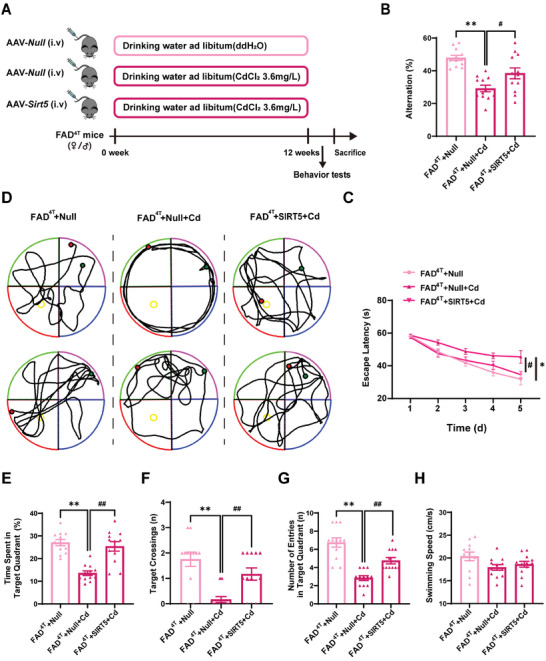
SIRT5 overexpression ameliorates Cd‐induced exacerbation of learning and memory deficits in FAD^4T^ mice. All the FAD^4T^ mice were intravenously injected with AAV‐*Null* or AAV‐*Sirt5* and exposed or not exposed to 3.6 mg L^−1^ CdCl_2_ for 12 weeks. A) Timeline depicting the experimental treatment for each group. B) Correct alterations in the Y‐maze test. *n* = 12 mice/group. C) Escape latency to reach the hidden platform in the Morris water maze. *n* = 12 mice/group. D) Representative track images in the Morris water maze. The results of the test included the time spent in the platform quadrant E), the number of crossings to the platform location F), the number of entries in the platform quadrant G), and the swimming speed of the FAD^4T^ mice (H). *n* = 12 mice/group. ^*^ *p* < 0.05 and ^**^ *p* < 0.01 versus the FAD^4T^+Null group; ^#^ *p* < 0.05 and ^##^ *p* < 0.01 versus the FAD^4T^+Null+Cd group.

Cd, an environmental factor, is one of the potential risks associated with the development of AD.^[^ ^7^ ^]^ FAD^4T^ mice, which serve as a reliable AD model and express accelerated amyloid plaques (1.5 months) accompanied by memory deficits (5 months),^[^ ^43^ ^]^ were used to evaluate the potential therapeutic efficacy of SIRT5 in this study. To further explore the neuroprotective effects of SIRT5 in vivo, adeno‐associated virus (AAV)‐*Sirt5* or the control (*Null*) were delivered to FAD^4T^ mice via tail vein injection, and the mice were then exposed to Cd (3.6 mg L^−1^) in drinking water provided ad libitum for 12 weeks (**Figure** **7**A). Notably, no obvious differences in mouse body weight or mean daily intake of drinking water among the 3 groups during the 12‐week period (Figure S9, Supporting Information). We found that overexpression of SIRT5 effectively reversed the decrease in the SIRT5 levels induced by Cd in the cortex and hippocampus of FAD^4T^ transgenic mice (Figure S10, Supporting Information). Subsequently, the mice were subjected to behavioral assessments. The Y‐maze test indicated that overexpression of SIRT5 apparently mitigated the detrimental impact of Cd exposure on spatial memory but did not disturb the locomotor activities of mice, as manifested by a decrease in the rate of spontaneous alternations and similar total distance of mice movement (Figure 7B; Figure S11, Supporting Information). In comparison to those in the control group, the Cd‐induced FAD^4T^ mice exhibited significant impairments in spatial learning and memory, as evidenced by a prolonged escape latency during the training trials, reductions in the time and frequency spent in and entering the target quadrant, and a decreased number of crossings over the platform (Figure 7C–G). However, SIRT5 overexpression markedly rescued these defects in FAD^4T^ mice induced by Cd (Figure 7C–G). Notably, the swimming speed did not significantly differ among these mice (Figure 7H). These results provide compelling evidence showing that SIRT5 represents a promising therapeutic target for mitigating spatial learning and memory impairments in Cd‐exposed FAD^4T^ mice.

**Figure 8:**
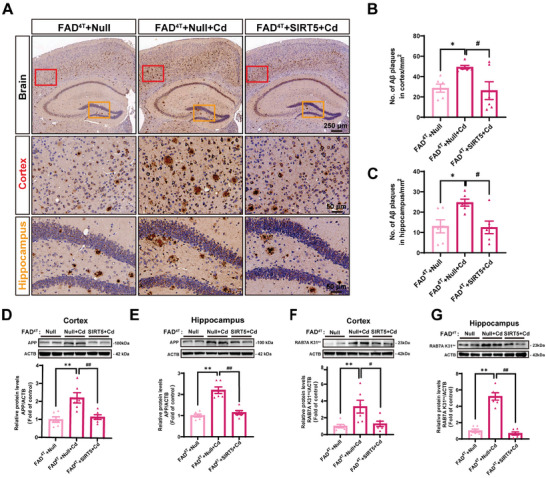
SIRT5 overexpression reduces β‐amyloid pathology and the RAB7A K31^su^ levels in Cd‐exposed FAD^4T^ mice. All the FAD^4T^ mice were intravenously injected with AAV‐*Null* or AAV‐*Sirt5* and exposed or not exposed to 3.6 mg L^−1^ CdCl_2_ for 12 weeks. A) Representative images of β‐amyloid staining in the cortex and hippocampus. Scale bar for the brain: 250 µm; scale bar for the cortex/hippocampus: 50 µm. Statistical analysis of Aβ plaques in the cortex B) and hippocampus C). *n* = 6 mice/group. Representative immunoblot and quantification of APP in the cortex D) and hippocampus E) of FAD^4T^ mice. *n* = 6 mice/group. Representative immunoblot and quantification of RAB7A K31^su^ in the cortex F) and hippocampus G) of FAD^4T^ mice. *n* = 6 mice/group. ^*^ *p* < 0.05 and ^**^ *p* < 0.01 versus the FAD^4T^+Null group; ^#^ *p* < 0.05 and ^##^ *p* < 0.01 versus the FAD^4T^+Null+Cd group.

Notably, β‐amyloid deposition is considered one of the most crucial pathological features of AD.^[^ ^44^ ^]^ An anti‐Aβ antibody was used to evaluate the Aβ burden in FAD^4T^ mice, and the Aβ plaques were quantified. We revealed that exposure to Cd clearly elevated the presence of Aβ plaques in the cortex and hippocampus of FAD^4T^ mice (**Figure** **8**A–C). Conversely, mice with SIRT5 overexpression demonstrated a noticeable reduction in the Aβ burden within the brain (Figure 8A–C). These similar results were supported by the alterations in APP expression in the cortex and hippocampus of FAD^4T^ mice (Figure 8D,E). Furthermore, SIRT5 overexpression apparently alleviated the Cd‐induced elevation in the RAB7A K31^su^ levels in the cortex and hippocampus of FAD^4T^ mice (Figure 8F,G).

**Figure 9:**
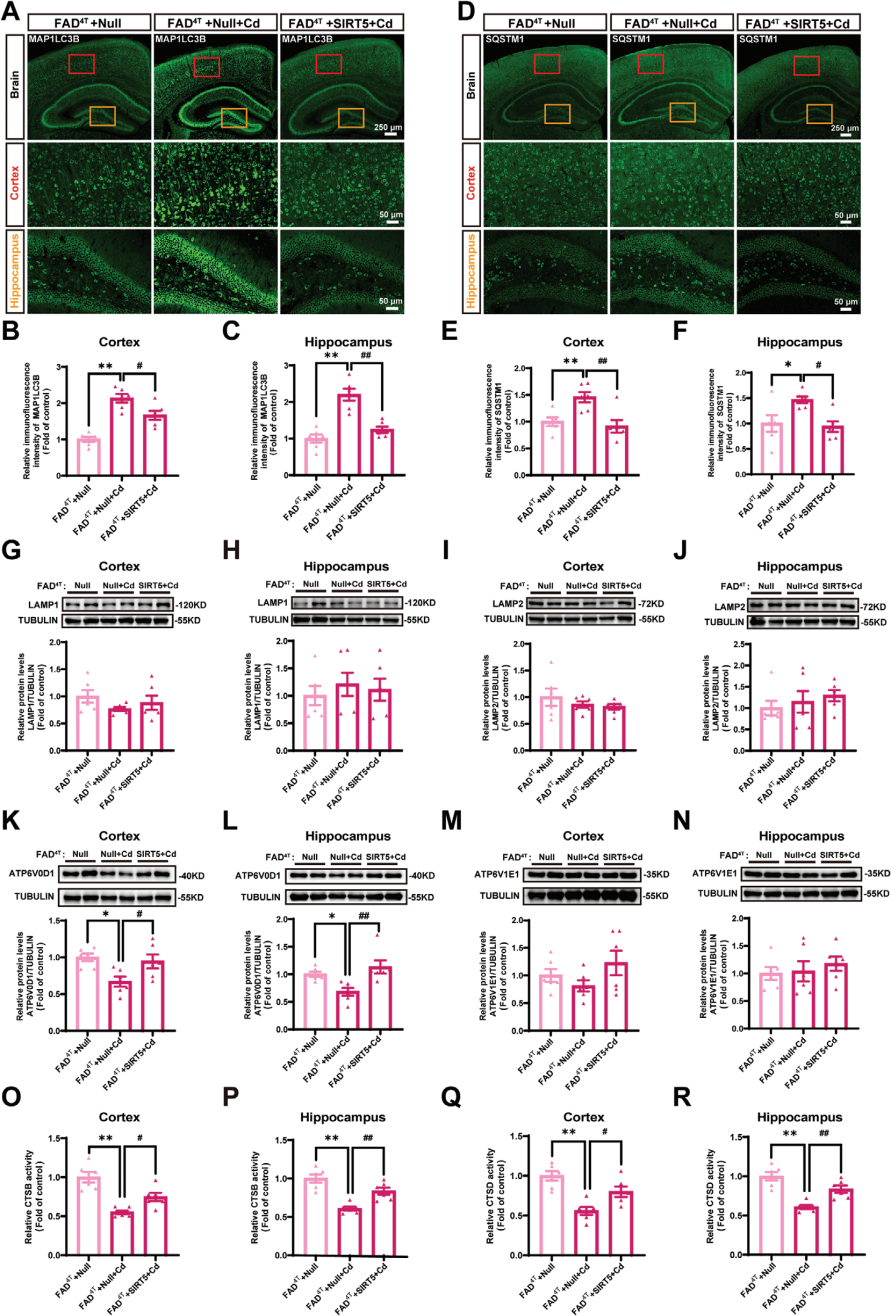
SIRT5 overexpression alleviates autophagic flux blockade and lysosomal dysfunction in Cd‐exposed FAD^4T^ mice. All the FAD^4T^ mice were intravenously injected with AAV‐*Null* or AAV‐*Sirt5* and exposed or not exposed to 3.6 mg L^−1^ CdCl_2_ for 12 weeks; n = 6 mice/group. Representative immunofluorescence images A) and relative fluorescence intensity of MAP1LC3B in the cortex B) and hippocampus C) of FAD^4T^ mice. Scale bar for the brain: 250 µm; scale bar for the cortex/hippocampus: 50 µm. Representative immunofluorescence images D) and relative fluorescence intensity of SQSTM1 in the cortex E) and hippocampus F) of FAD^4T^ mice. Scale bar for the brain: 250 µm; scale bar for the cortex/hippocampus: 50 µm. Representative immunoblot and quantification of LAMP1 and LAMP2 in the cortex G and I) and hippocampus H and J) of FAD^4T^ mice. Representative immunoblot and quantification of ATP6V0D1 and ATP6V1E1 in the cortex (K and M) and hippocampus (L and N) of FAD^4T^ mice. Relative CTSB and CTSD activities in the cortex (O and Q) and hippocampus (P and R) of FAD^4T^ mice. ^*^ *p* < 0.05 and ^**^ *p* < 0.01 versus the FAD^4T^+Null group; ^#^ *p* < 0.05 and ^##^ *p* < 0.01 versus the FAD^4T^+Null+Cd group.

Subsequently, the alterations in the autophagy process in each group were evaluated. As anticipated, immunohistochemical staining indicated that overexpression of SIRT5 clearly mitigated the heightened levels of MAP1LC3B and SQSTM1 triggered by Cd in the cortex and hippocampus of FAD^4T^ mice (**Figure** **9**A–F). However, there was no disparity observation in the levels of LAMP1 or LAMP2 in the brains of the mice, which was not precisely aligned with the previous in vitro findings (Figure 9G–J). ATP6V0D1 (ATPase H^+^ transporting lysosomal V0 subunit D1) and ATP6V1E1 (ATPase H^+^ transporting lysosomal V1 subunit E1) are members of the vacuolar H^+^‐ATPase family and play crucial roles in maintaining an acidic lysosomal environment.^[^ ^40^, ^45^ ^]^ The present study revealed that the levels of ATP6V0D1 were intensively decreased after exposure to Cd and could be restored through overexpression of SIRT5 in the cortex and hippocampus of FAD^4T^ mice, although no obvious alterations in the levels of ATP6V1E1 were detected (Figure 9K–N). Additionally, the activities of CTSB and CTSD were intensively dampened by Cd treatment, but this reduction could be reversed by SIRT5 overexpression in the cortex and hippocampus of FAD^4T^ mice (Figure 9O–R). These results emphasize the potential neuroprotective effect of the SIRT5‐RAB7A axis in vivo.

### 2.7 The *SIRT5* Levels are Mainly Downregulated in the Brain Neurons of AD Patients

**Figure 10:**
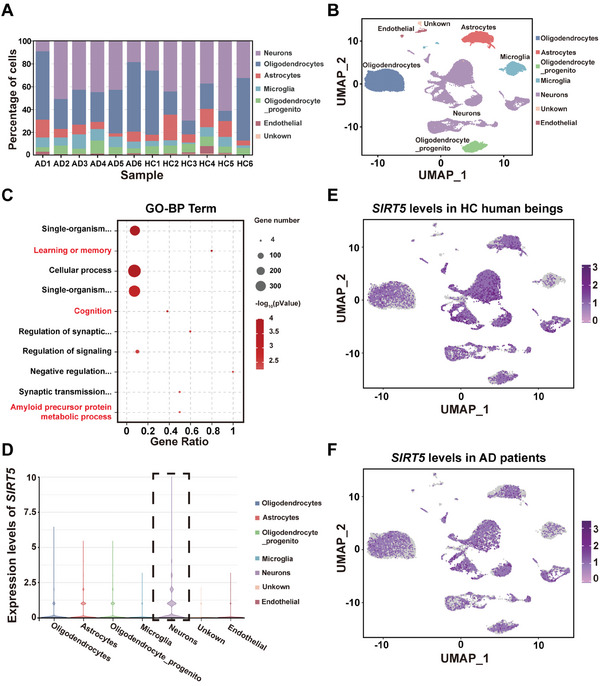
The *SIRT5* levels are clearly lower in the brain neurons of AD patients than in those of healthy controls (HCs). A) Bar plot showing the relative percentages of different cell types in the brains of AD patients and HCs. B) UMAP plot showing clusters of annotated cells. C) Results of the GO enrichment analysis of the DEGs in the neurons of AD patients compared with those of HCs. The analysis was based on the biological process (BP) category. *n* = 6/group. D) Violin diagram indicating that the SIRT5 levels in neurons were greater than those in other cell types. *n* = 6/group. UMAP plot showing the differentially expressed gene *SIRT5* in the brains of HCs E) and AD patients F). n = 6/group.

To determine the clinical relevance of SIRT5, we downloaded the raw single‐nucleus RNA sequencing (snRNA‐seq) data from postmortem frozen human brain tissues of patients with AD or healthy controls (HCs) in the GSE188545 dataset (Table S5, Supporting Information).^[^ ^46^ ^]^ After cell filtering, a total of 75 052 single‐cell transcriptomes were obtained, and medians of 1901 and 2316 genes were detected in the AD and HC groups, respectively (Figure S12, Supporting Information). We further clustered the cell populations and annotated the major cell types in the human brain by examining the expression patterns of known gene markers, including those of neurons (*NRGN*, *CAMK2A*, and *GAD1*), astrocytes (*AQP4* and *GFAP*), microglia (*CSF1R*, *CD74*, and *C3*), oligodendrocytes (*MBP*), oligodendrocyte precursor cells (*VCAN*), and endothelial cells (*FLT1* and *CLDN5*) (**Figure** **10**A,B). Quantitative data with a *p*‐value <0.05 and a fold change >1.28 or <0.77 were considered as differentially expressed genes (DEGs).^[^ ^47^ ^]^ A GO analysis was performed to further assess the potential influence of DEGs in neurons. We found that DEGs in neurons were significantly enriched in terms relevant to AD, such as learning or memory, cognition and amyloid precursor protein metabolic process (Figure 10C). Intriguingly, a violin diagram indicated that the expression level of *SIRT5* in neurons was greater than that in other cell types (Figure 10D). Importantly, compared with those in the HCs, the levels of *SIRT5* were decreased in neurons but not in other cell clusters in the brains of AD patients (Figure 10E,F).

## 3 Discussion

Recently, Cd exposure serves as a pivotal environmental risk factor for aggravating AD progression and poor outcomes, but the underlying mechanism has not been elucidated. In this study, we first revealed that i) Cd exposure triggered autophagic flux blockade and exacerbated AD‐like pathology; ii) SIRT5 desuccinylated and activated RAB7A at the K31 residue to rescue the Cd‐provoked AD progression by maintaining the autophagic flux; and iii) the SIRT5 levels were mainly decreased in the neurons of AD patients, which confirmed the clinical relevance of SIRT5 in AD progression.

AD, a degenerative neurological disorder, is becoming increasingly prevalent and is regarded as one of the most debilitating conditions, not only for the affected individuals but also for their families and caregivers.^[^ ^48^ ^]^ Emerging reports indicate that environmental factors are attributed to the progression of multiple diseases.^[^ ^49^ ^]^ Understanding the environmental factors that contribute to the risk of AD development can have significant impacts on the prevention and treatment strategies.^[^ ^50^ ^]^ Since Cd, a nonessential metal, has been recognized for its neurotoxic properties even at low levels, it is of particular interest due to its widespread population exposure and linkage with AD progression.^[^ ^8^ ^a,^ ^51^ ^]^ In this study, we orally administered Cd to FAD^4T^ mice at a dose of 3.6 mg L^−1^, which was 1/120 of the median lethal dose identified in our previous study, for 12 weeks.^[^ ^52^ ^]^ This dose and exposure model aligns closely with the findings of other studies. Wang et al. discovered that after 13 weeks of exposure to 3 mg L^−1^ Cd, the average peak blood Cd concentration was ≈2.25 µg L^−1^,^[^ ^53^ ^]^ similar to the blood concentration in male adults in China (1.34, 0.38–2.88 µg L^−1^).^[^ ^54^ ^]^ Hence, the dosage of Cd applied in our research was consistent with the level of Cd exposure observed in human subjects, thereby holding significant value in establishing a direct correlation and elucidating the potential mechanism underlying the relationship between environmental Cd exposure and AD.

Mounting evidence indicates that autophagy, an evolutionarily conserved process for eliminating aggregated and misfolded proteins, is extensively involved in aspects of the pathogenesis of AD, such as Aβ metabolism and secretion.^[^ ^55^ ^]^ In addition, defective autophagy contributes to AD progression.^[^ ^55^ ^]^ Autophagy is also deemed a pivotal self‐protective mechanism for eukaryotes against heavy metal toxicity, and autophagy dysfunction plays a key role in Cd‐induced cytotoxicity.^[^ ^56^ ^]^ In line with previous reports, we also found that acute exposure to Cd hindered autophagic flux and suppressed the expression of transcription factor EB (TFEB), a key regulator of lysosomes and autophagy, which triggers neurotoxicity.^[^ ^57^ ^]^ Intriguingly, the current study further proved the explicit linkage of Cd‐provoked autophagic flux blockade and Aβ deposition with cognitive decline in AD model (FAD^4T^) mice. Notably, our study specifically clarified the Cd‐induced obstacle in the fusion between autophagosomes and lysosomes and impaired lysosomal function, as indicated by decreased activity of CTSB in vitro and in vivo, which is in keeping with previous findings on the anti‐amyloidogenic functions of CTSB.^[^ ^58^ ^]^ Of note, these findings imply that the advanced stage of autophagic progression is prone to damage during the Cd‐induced worsening of AD pathology.

SIRT5 modulates various crucial biological processes that have been implicated in multiple diseases, such as cancer and neurological, cardiovascular, and metabolic disorders.^[^ ^59^ ^]^ In particular, given the diminished levels of SIRT5 in various neurodegenerative disorders, SIRT5 has been deemed a potential neuroprotective factor.^[^ ^60^ ^]^ Liu et al. reported that SIRT5 protects against motor impairment and dopaminergic degeneration in a mouse model of PD induced by MPTP.^[^ ^23^ ^b]^ Li et al. reported that SIRT5 deficiency elevates the susceptibility to seizures induced by kainate and exacerbates neurodegeneration in the hippocampus of mice.^[^ ^61^ ^]^ Consistent with previous studies, we analyzed the snRNA‐seq data of AD and control brains and found that the SIRT5 levels were markedly decreased in the neurons of AD brains. In addition, the SIRT5 was robustly weakened in the cortex and hippocampus of Cd‐induced AD model mice and Cd‐treated Neuro‐2a cells. Importantly, SIRT5 overexpression profoundly counteracted the Cd‐evoked AD‐like pathology in vivo and in vitro. Given the diminished levels of SIRT5 in various neurodegenerative disorders and the beneficial restorative effects observed upon the reintroduction of SIRT5 in both our research and other studies, there is growing interest in the targeting of SIRT5 as a therapeutic strategy for Cd‐induced AD‐like pathology. Several studies indicated that transcription factor‐downstream gene cascades play a crucial role in Cd‐triggered toxicity, activating various pathways such as autophagic dysregulation, endoplasmic reticulum stress, and apoptosis, ultimately leading to cellular damage.^[^ ^62^ ^]^ Additionally, differentially expressed proteins identified from quantitative proteomics were utilized for upstream regulator analysis based on the IPA algorithm,^[^ ^63^ ^]^ suggesting that peroxisome proliferator‐activated receptor alpha (PPARA), a crucial transcriptional regulator, was identified as a potential upstream regulator of SIRT5 (Figure S13, Supporting Information). PPARA has been implicated in the modulation of autophagy‐lysosomal function.^[^ ^64^ ^]^ It was demonstrated that Cd impaired the autophagic flux by inhibiting PPARA expression, contributing to nonalcoholic fatty liver disease as well as hepatotoxicity.^[^ ^65^ ^]^ Considering these findings, we posit that Cd exposure may inhibit the levels of SIRT5 by disturbing PPARA in Neuro‐2a cells. Further comprehensive exploration is required to unmask the intrinsic mechanism governing this interaction.

SIRT5 has emerged as a unique deacetylase that possesses diverse enzymatic capabilities for the hydrolysis of negatively charged lysine residues to regulate the autophagy machinery, including succinylation, malonylation, and glutarylation.^[^ ^66^ ^]^ Zhang et al. reported that SIRT5‐induced autophagic flux is dependent on the desuccinylation of OPTN K108^su^, which plays a protective role in the function of retinal ganglion cells in diabetic retinopathy.^[^ ^67^ ^]^ Additionally, the SIRT5‐induced deacetylation of lactate dehydrogenase B (LDHB) has been found to promote autophagy in colorectal cancer.^[^ ^68^ ^]^ In this study, SIRT5 overexpression apparently facilitated the blockade of autophagic flux induced by Cd in vivo and in vitro, but the inactive mutant SIRT5‐H158Y had no protective effect, suggesting that PTMs modulated by SIRT5 are an essential target in Cd‐induced neurotoxic effect. Notably, the current study discovered that exposure to Cd significantly elevated the levels of succinylation but did not affect the levels of malonylation or glutarylation. Considering the potential competition between various modifications, Cd dampened SIRT5 activity, which specifically promoted the succinylation of multiple proteins may be a particular manner of the epigenetic alterations induced by environmental pollutants.

Furthermore, our work first identified a pivotal downstream target of SIRT5, namely, a single succinylation site in RAB7A that was strongly elevated by Cd in vivo and in vitro. RAB7A is a critical GTPase that plays an essential role in the regulation of late endocytic trafficking and maturation, including the maturation of autophagosomes and their fusion with lysosomes to form autolysosomes.^[^ ^69^ ^]^ Emerging reports indicate that PTMs are involved in the regulation of RAB7A activity and function. Heo et al. reported that tank‐binding kinase 1 (TBK1) phosphorylates the S72 residue of RAB7A and enhances its activity, which promotes mitophagy.^[^ ^37^ ^]^ Gayatree Mohapatra et al. revealed that the SUMOylation process switches RAB7A from a nonfunctional to a functional state, controlling *Salmonella* Typhimurium intracellular life and pathogenesis.^[^ ^70^ ^]^ Lysine succinylation serves as a novel type of PTM; owing to its negatively charged nature and large size, lysine succinylation is more prone to exert substantial impacts on protein activity, structure, and stability than other modifications.^[^ ^71^ ^]^ Teng et al. reported that desuccinylation of mitochondrial malic enzyme 2 (ME2) at Lys346 promoted its enzymatic activity, thus enhancing mitochondrial respiration and antagonizing glutamine deprivation in colorectal cancer cells.^[^ ^72^ ^]^ Yang et al. reported that desuccinylation of Lys280 on SHMT2 resulted in its activation, leading to increased serine catabolism and ultimately promoting tumor cell growth.^[^ ^73^ ^]^ Consistent with previous reports, our study revealed that mutant RAB7A_K31R_, which mimics desuccinylated RAB7A, strongly promoted the recruitment of the effector RILP to facilitate the impairments of autophagosome‐lysosome fusion and lysosome function induced by Cd, thus supporting the survival of Neuro‐2a cells; however, the mutant RAB7A_K31E_ had no protective effect. Our data innovatively highlighted the crucial role of the desuccinylation modification of RAB7A, which promotes RAB7A activity and ultimately regulates the Cd‐mediated inhibition of autophagic flux in nerve cells. In addition to small GTPases, the fusion of autophagosomes and lysosomes requires the involvement of soluble N‐ethylmaleimide‐sensitive factor attachment protein receptors (SNAREs) and tethering complexes.^[^ ^74^ ^]^ Syntaxin 17 (STX17), an essential player of the SNARE systems primarily localized in autophagosomes, has been demonstrated to facilitate the recruitment of the RAB7A effector, ectopic P‐granules autophagy protein 5 homolog (EPG5), and the homotypic fusion and protein sorting (HOPS)‐tethering complex to autophagosomes in anticipation of fusion with lysosomes.^[^ ^75^ ^]^ Notably, Cd exposure markedly downregulated the expression levels of HOPS subunit vacuolar protein sorting 41 (VPS41), which impeded the autophagosome‐lysosome fusion and led to autophagic dysfunction in rat cerebral cortical neurons and mouse hepatocytes.^[^ ^76^ ^]^ Duan et al. reported Cd exposure significantly inhibited the colocalization between STX17 and MAP1LC3B, thereby disrupting the fusion of autophagosomes with lysosomes in rat liver cells.^[^ ^77^ ^]^ Above all, these findings suggest that Cd has a significant impact on the dominant linker that promotes autophagosomal and lysosomal fusion. Further investigation is warranted to elucidate the underlying mechanisms and evaluate the potential of targeting the dominant linker as a therapeutic strategy for Cd‐associated brain degeneration.

Some limitations are present in this study. SIRT5 may have multiple downstream targets involved in the pathogenesis of AD. According to the results of a lysine succinylome analysis, numerous hypersuccinylated proteins enriched in autophagy‐related pathways and the AD pathway warrant further investigation. Additionally, due to limitations in the availability of the anti‐RAB7A‐succinyl‐K31 antibody, we only employed a Western blot analysis, which resulted in a lack of immunofluorescence detection in this study.

## 4 Conclusion

**Figure 11:**
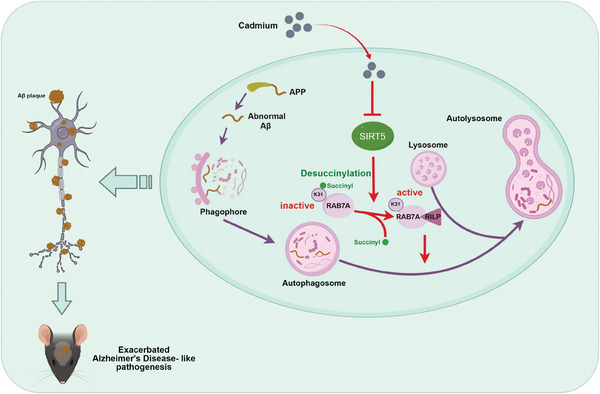
Schematic diagram indicating that SIRT5‐mediated RAB7A desuccinylation antagonizes Cd‐induced AD‐like pathogenesis by maintaining autophagic flux.

In summary, this study provides the first indication that SIRT5 desuccinylated RAB7A at the Lys31 residue, which facilitated its activity. This activation of RAB7A served as a protective mechanism against autophagic flux blockade, ultimately mitigating the Cd‐induced exacerbation of AD‐like pathology and cognitive decline (**Figure** **11**). These findings enhance the understanding of the intricate interplay between genes and the environment in AD pathogenesis. In addition, our research identified potential cellular therapeutic targets for the prevention and management of environmental contaminant‐associated brain degeneration.

## 5 Experimental Section

### Cell Culture and CdCl_2_ Treatment

Mouse neuroblastoma cells (Neuro‐2a cells) were obtained from the Cell Bank of the Institute of Biochemistry and Cell Biology (Shanghai, China, TCM29) and were treated with 0, 1, 2, or 4 µM CdCl_2_ for 72 h as reported previously.^[^ ^40^ ^]^ Neuro‐2a cells were cultured in DMEM/H (Gibco, C11995500BT) supplemented with 10% foetal bovine serum (AUSGENEX, FBS500‐S) and 1% penicillin/streptomycin (Beyotime, C0222) at 37 °C in an atmosphere of 5% CO_2_.^[^ ^40^ ^]^ A stock solution of CdCl_2_ (Sigma‒Aldrich, 202 908) was prepared using distilled deionized water and diluted with medium accordingly. In addition, 25 and 37.5 µM chloroquine (CQ, MedChemExpress, HY‐17589A) and 1 mM 3‐methyladenine (3‐MA, MedChemExpress, HY‐19312) were used in this study.

### Cytotoxicity Detection

To detect cell proliferation, Neuro‐2a cells were inoculated at a density of 3.5×10^3^ cells per well in 96‐well ImageLock plates (Essen BioScience, USA). After treatment, the plate was placed into an Incucyte ZOOM apparatus, and images of the cells were recorded. Cell confluence was analyzed using the Incucyte ZOOM Live Cell Analysis System (Essen BioScience, USA) as previously described.^[^ ^78^ ^]^

### Plasmid Construction and Transfection

Wild‐type SIRT5 (SIRT5‐WT), enzymatically inactive SIRT5 containing a mutation at amino acid 158 (H to Y) (SIRT5‐H158Y),^[^ ^68^ ^]^ and mutant RAB7A containing a mutation at amino acid 31 [K to R (RAB7A_K31R_) or K to E (RAB7A_K31E_)] were cloned and inserted into the pcDNA3.1 vector with a 3×Flag tag by GenePharma (Shanghai, China). All the expression constructs were verified by Sanger sequencing. All the plasmids were transfected using X‐tremeGENE HP DNA Transfection Reagent (Roche, 6 366 236 001),^[^ ^79^ ^]^ and the empty pcDNA 3.1 vector served as a negative control (Vector).

### siRNA Transfection

ATG5 was knocked down in Neura‐2a cells using small interfering RNAs (siRNAs) (Santa Cruz, sc‐41446, USA) transfected with Lipofectamine RNAiMAX (Thermo Fisher, 13 778 075, USA).^[^ ^80^ ^]^ A scrambled sequence (Santa Cruz, sc‐37007, USA) was used as a negative control (NC).

### Autophagic Flux Monitoring

The GFP‐LC3B plasmid was transfected into cells to monitor autophagosome formation, and quantification was performed as previously described.^[^ ^40^ ^]^ Premo Autophagy Tandem Sensor RFP‐GFP‐LC3B (Invitrogen, P36239) was used to monitor the transformation of an autophagosome (with neutral pH) to an autolysosome (with an acidic pH).^[^ ^40^ ^]^ Neuro‐2a cells were seeded on glass‐bottom dishes and treated with Premo Autophagy Tandem Sensor for 24 h. The Neuro‐2a cells were then exposed to Cd for an additional 72 h. The samples were detected using a confocal laser scanning microscope (Leica, Stellaris 5, Germany) equipped with a 63× oil immersion objective.

### Tandem Mass Tag (TMT)‐Based Quantitative Proteomic Analysis

A cohort of 5×10^6^ Neuro‐2a cells per group was subjected to treatment with or without 4 µM CdCl_2_ for 72 h. Subsequently, the cells were lysed in SDT buffer for TMT‐based quantitative proteomic analysis, which was performed by Applied Protein Technology (Shanghai, China) as described in the previous study.^[^ ^79^ ^]^ The dataset identifier for the mass spectrometry proteomics data deposited in the ProteomeXchange Consortium was PXD030587.^[^ ^79^ ^]^

### LysoSensor Green DND‐189 Detection

The lysosomal pH was determined by incubating cells with 1 µM LysoSensor Green DND‐189 (Invitrogen, L7535) for 5 min at 37 °C as previously reported.^[^ ^40^ ^]^ The fluorescence intensity was quantified by a microplate reader (Infinite M200) with 485‐nm excitation and 530‐nm emission filters.

### CTSB and CTSD Activity Detection

CTSB activity and CTSD activity were determined using a CTSB activity fluorometric assay kit (BioVision, K140‐100) and a CTSD activity fluorometric assay kit (BioVision, K143‐100), respectively, as previously reported.^[^ ^40^ ^]^ Cell and brain samples (from the cortex and hippocampus, respectively) were collected with the chilled lysis buffer supplied with the kit. The tissue samples were obtained and homogenized using TissueLyser II (QIAGEN, 85300). After centrifugation, the supernatant was used for the detection of CTSB or CTSD activity. CTSB activity was measured by a microplate reader (Infinite M200) with 400‐nm excitation and 505‐nm emission filters, and CTSD activity was measured by a microplate reader (Infinite M200) with 328‐nm excitation and 460‐nm emission filters. All activities of the samples were normalized to the protein concentrations.^[^ ^40^ ^]^

### 4D‐label‐free Quantitative Proteomics of Succinylation

1 × 10^7^ Neuro‐2a cells treated with or without CdCl_2_ treatment for 72 h were collected (*n* = 4), and 4D label‐free succinylation quantitative proteomic analysis was performed by Jingjie PTM BioLab Co., Ltd. (China, Hangzhou).^[^ ^81^ ^]^ Protein samples were obtained and quantified using a BCA kit. The proteins were digested by trypsin, and the peptides were desalted on a C18 SPE column. The tryptic peptides were incubated with anti‐succinyl lysine antibody beads (PTM Bio, PTM‐402) for protein PTM enrichment, and the resulting peptides were desalted with C18 ZipTips (Millipore). These peptides were subsequently subjected to LC‒MS/MS analysis. The MS/MS data were processed with the MaxQuant search engine (v.1.6.15.0). A bioinformatics analysis was then performed. The dataset identifier for the mass spectrometry succinylation quantitative proteomics data deposited in the ProteomeXchange Consortium was PXD048158.

### Bioinformatics Analysis for Quantitative Proteomic Detection

KEGG and IPA analyses were also conducted to examine the pathways enriched in the differentially expressed proteins identified from the TMT‐based quantitative proteomic analysis of Neuro‐2a cells treated or not treated with 4 µM CdCl_2_ for 72 h, as previously reported.^[^ ^79^ ^]^

### Development and Characterization of the Rabbit Anti‐RAB7A‐succinyl‐K31 Antibody

The rabbit polyclonal antibody that specifically recognizes succinyl at RAB7A K31 was produced by Jingjie PTM BioLab Co., Ltd. (China, Hangzhou). Briefly, 2 succinyl‐modified peptides, and one nonmodified control peptide were synthesized and confirmed by MS detection (Figure S6 and Table S1, Supporting Information). Two succinyl‐modified peptides were used to immunosuppress 6 rabbits. The samples were then subjected to serum screening by ELISA and Western blotting, and the results were positive (rabbit‐R4/5/6) and used for purification (Figure S7 and Table S2, Supporting Information). The purified Ab4/5/6 antibodies were confirmed by ELISA (Table S3, Supporting Information). The specificity of Ab5 was verified by Dot blotting assay (Figure 4F). Ab5 was ultimately selected for RAB7A K31^su^ detection by Western blotting.

### Dot Blotting

Dot blot assay was conducted with an ECL chemiluminescence kit (Beyotime, BeyoECL Moon) as previously reported.^[^ ^67^ ^]^ Different doses of modified peptides and non‐modified peptides were fixed on the PVDF membranes. After incubation, enzyme‐conjugated secondary antibody and chemiluminescence substrate were applied to detect the binding between the polypeptide and anti‐RAB7A‐succinyl‐K31 antibodies. Comprehensive information regarding the antibodies employed can be found in Table S6 (Supporting Information).

### Immunocytochemistry Assay

Following treatment, the Neuro‐2a cells were fixed in 4% paraformaldehyde at room temperature for 20 min (PFA, Sigma‒Aldrich, 16 005). The nuclei of the cells were visualized using DAPI staining solution (Beyotime, C1005). The cells were examined using a confocal microscope with a 63× objective, and the fluorescence intensity was assessed in 4 distinct fields per dish (Leica Stellaris 5, Germany). The comprehensive information of primary and secondary antibodies is listed in Table S6 (Supporting Information).

### Western Blotting

Following treatment, cells or tissues were harvested and subjected to lysis using RIPA lysis buffer (Beyotime, P0013B) supplemented with a mixture of protease inhibitors (Roche, USA) for subsequent Western blot analysis.^[^ ^82^ ^]^ Comprehensive information regarding the antibodies employed can be found in Table S6 (Supporting Information).

### Molecular Docking

Molecular docking analysis was conducted using the Docking Web Server (GRAMM) and AutoDockTools‐1.5.7 software.^[^ ^83^ ^]^ The crystal structure of SIRT5, as predicted by the AlphaFold protein structure database, was docked with the crystal structure of RAB7A obtained from the PubChem protein database (PDB ID: 5JRH). The regularized protein was utilized to identify the essential amino acids within the predicted binding site.

### Microscale Thermophoresis Binding Assay

Microscale thermophoresis (MST) was performed by TopScience Co., Ltd (Shanghai, China) as previously described.^[^ ^84^ ^]^ Recombinant mouse proteins (SIRT5 or RAB7A) were purified from 293F cells (Figure S8, Supporting Information). MST assays were performed with a Monolith NT.115 instrument (NanoTemper, Germany), and the *K*_D_ values were determined with Mono‐Temper analysis software (NanoTemper, Germany).

### Surface Plasmon Resonance (SPR) Measurement

SPR assays were performed by TopScience Co., Ltd (Shanghai, China) as previously described.^[^ ^85^ ^]^ Recombinant mouse proteins (SIRT5 or RAB7A) were purified from 293F cells (Figure S8, Supporting Information). The RAB7A protein was immobilized on a CM5 sensor chip (Cytiva, BR‐1005‐30). A twofold variable concentration dilution series of SIRT5 was injected into the chip at a flow rate of 10 µl min^−1^ for 60 s, followed by dissociation (90 s). The experiments were conducted on a BIAcore T200 instrument (v.2.0 GE Healthcare, USA), and the *K*_D_ values were determined based on the 1:1 Langmuir binding model using BIAcore T200 Evaluation software (v.2.0 GE Healthcare, USA) following the manufacturer's guidelines.

### Animal Models

The animal experiments conducted in this study were approved by the Animal Care and Use Committee of the Army Medical University (AMUWEC20230067). The FAD^4T^ [B6/JGpt‐Tg (Thy‐APP/Thy‐PSEN1)5/Gpt] transgenic mouse was a multi transgenic AD model generated by GemPharmatech Co., Ltd. (Nanjing, China), which expresses the Swedish and Indiana mutated APP genes and the PSEN1 gene with M146V and L286V mutations.^[^ ^43^ ^]^ The FAD^4T^ mice (aged 6.5 weeks) were acclimatized for one week in a room with a 12‐h light/dark cycle and were provided standard laboratory food and water. Subsequently, the FAD^4T^ mice were randomly allocated into 3 groups, with an equal distribution of male and female individuals within each group (*n* = 12 mice/group). An AAV PHP.eB overexpressing the mouse *Sirt5* gene (pAAV‐Syn‐*Sirt5*‐3×FLAG‐tWPA) and control (*Null*) gene (pAAV‐Syn‐MCS‐3×FLAG‐tWPA) were developed by OBiO Technology (Shanghai, China).^[^ ^86^ ^]^ The FAD^4T^ mice in the AAV‐*Null+*Cd or AAV‐*Null* group were intravenously injected with 2 × 10^11^ AAV‐*Null* dissolved in saline,^[^ ^40^ ^]^ and 3 days later, the mice had ad libitum access to purified water supplemented with or without 3.6 mg L^−1^ CdCl_2_ for 12 weeks. Moreover, the FAD^4T^ mice were intravenously injected with 2 × 10^11^ AAV‐*Sirt5* dissolved in saline, and 3 days later, the mice had ad libitum access to drinking water with 3.6 mg L^−1^ CdCl_2_ for 12 weeks (AAV‐*Sirt5*+Cd). After treatment, the mice were subjected to behavioral tests. The mice were transcardially perfused with 0.9% saline under pentobarbital anesthesia and then perfused with 4% PFA in PBS (*n* = 6 mice/group). Subsequently, the brains were extracted and fixed in 4% PFA until use. Moreover, cortical and hippocampal tissues were obtained from a separate cohort of euthanized mice, immediately frozen in liquid nitrogen, and subsequently stored at −80 °C until analysis (*n* = 6 mice/group).

### Y‐Maze Test

After Cd treatment, the Y‐maze test was conducted as previously described.^[^ ^87^ ^]^ The Y‐maze consisted of 3 nontransparent arms with a length of 30 cm, a width of 8 cm, and 15 cm high walls positioned at 120° angles from each other. The mice were placed in the center of the maze and allowed to explore the arms spontaneously for a duration of 8 min. Video analysis software (SMART 3.0) was utilized to track the animals and record the data. Spontaneous alternation was defined as consecutive entries into 3 different arms (e.g., BAC, CBA, or ACB but not ABA or BCB). After each test, the arms were cleaned extensively using 70% ethanol to eliminate any lingering odors. The calculation of the alternation percentage was automatically performed by SMART 3.0 according to the following formula: alternation percentage = (number of alternations)/ (total arm entries − 2) × 100.

### Morris Water Maze (MWM) Test

The MWM test was conducted in accordance with the previous study.^[^ ^79^ ^]^ In brief, a tank with a diameter of 120 cm and a depth of 50 cm was filled with opaque water maintained at a temperature of 21 ± 1 °C and was utilized for the experimental procedures. Within the tank, a 10‐cm diameter escape platform was positioned, with its top situated 1 cm below the water surface. The mice underwent training for a period of 5 days, and this training consisted of 4 trials per day lasting 60 s each, with 30‐min intertrial intervals. Failure to locate the platform within 60 s resulted in the animal being placed on the platform for 20 s. Twenty‐four hours after the final training session, the platform was dismantled, and the mouse underwent memory retention testing in a probe trial, during which the mouse was allowed to swim freely for a duration of 60 s. A video analysis system (SMART 3.0 software) was utilized to monitor the animals and capture the data, and the behavioral parameters were automatically computed.

### Immunohistochemistry and Immunocytochemistry Assays of Mouse Brain

Mouse brains were extracted, fixed overnight in 4% PFA, embedded in paraffin, and cut into 5‐µm‐thick coronal sections. The slides were subjected to deparaffinization, rehydration, antigen retrieval, H_2_O_2_ treatment, and washing. Finally, the nuclei of the cells were visualized using DAPI staining solution (Beyotime, C1005).^[^ ^79^ ^]^ The slides were observed and imaged using a digital pathological section scanner (Pannoramic MIDI, 3DHISTECH, Hungary).^[^ ^79^ ^]^ The primary and secondary antibodies employed in this study can be found in Table S6 (Supporting Information). The immunofluorescence intensity of MAP1LC3B and SQSTM1, as well as the quantification of Aβ plaques, in the cortex and hippocampus were assessed in a blinded manner using ImageJ Pro Plus software.^[^ ^88^ ^]^

### Single‐Nucleus RNA Sequencing (snRNA‐seq) Data Processing

snRNA‐seq data processing was performed by Gene Denovo Biotechnology (Guangzhou, China). In brief, snRNA‐seq data (GSE188545)^[^ ^46^ ^]^ of postmortem brain tissues from AD patients (*n* = 6) or healthy controls (HCs, *n* = 6) were downloaded from Gene Expression Omnibus (GEO) datasets, and the Seurat package in R (version 4.0.3) with R studio (version 1.3.1903) was used to process the data. The detailed information is listed in Table S5 (Supporting Information). The GSE188545 single‐nucleus library was generated on the 10 × Chromium platform (10 × Genomics) and sequenced on a NovaSeq 6000 S4 sequencer (Illumina). Cells were filtered based on the presence of >25% mitochondria‐related genes or the expression of more than 8500 genes. A total of 75052 cells were selected for subsequent analysis, and the variable features of each sample were examined following normalization. The Seurat function FindVariableFeatures was then utilized to merge the sample files with common anchors among variables. Gene Ontology (GO) enrichment analysis and all the visualization of the results were performed by Omicsmart (www.omicsmart.com).

### Statistical Analysis

The data were analyzed utilizing GraphPad Prism 9.0 software (GraphPad, USA) and were presented as the means ± SEMs. Comparisons between 2 experimental groups were statistically assessed using unpaired two‐tailed t‐tests or nonparametric Mann‒Whitney U tests, whereas the differences among multiple groups were evaluated by Ordinary one‐way analysis of variance (ANOVA) or nonparametric Kruskal‒Wallis ANOVA followed by Dunnett's multiple comparisons test. Each experiment was replicated a minimum of 3 times, and ^*^ *p* < 0.05 or ^**^ *p* < 0.01 was defined as indicating statistical significance.

## Conflict of Interest

The authors declare no conflict of interest.

## Author Contributions

P.D. and T.F. contributed equally to this work. H.P., Z.Y., Z.Z., and P.D. designed the project. P.D., T.F., P.G., Y.P., M.L., J.L., M.Q., R.H., L.W., M.L., L.Z., C.C., M.H., Y.L., Q.M., Y.L., L.T., J.X., and M.C. performed the experiments. P.D., T.F., and M.L. analyzed the data. P.D. and H.P. wrote the manuscript. H.P., Z.Y., Z.Z., and S.X. reviewed and edited the manuscript.

## Supporting information

Supporting Information

## Data Availability

The data that support the findings of this study are available from the corresponding author upon reasonable request.
