# Supplementary material for: SIRT5‐Mediated Desuccinylation of RAB7A Protects Against Cadmium‐Induced Alzheimer's Disease‐Like Pathology by Restoring Autophagic Flux
Source: Adv Sci (Weinh). 2024 Jun 5;11(30):2402030. doi: 10.1002/advs.202402030 (PMC11321632; doi:10.1002/advs.202402030)
Supplement: Supplementary file 1 — Supporting Information [file ADVS-11-2402030-s001.docx]

Supporting Information

# SIRT5-Mediated Desuccinylation of RAB7A Protects Against Cadmium-Induced Alzheimer's Disease-Like Pathology by Restoring Autophagic Flux

*Ping Deng, Tengfei Fan, Peng Gao, Yongchun Peng, Min Li, Jingdian Li, Mingke Qin, Rongrong Hao, Liting Wang, Min Li, Lei Zhang, Chunhai Chen, Mindi He, Yonghui Lu, Qinlong Ma, Yan Luo, Li Tian, Jia Xie, Mengyan Chen, Shangcheng Xu, Zhou Zhou*, Zhengping Yu*, and Huifeng Pi**


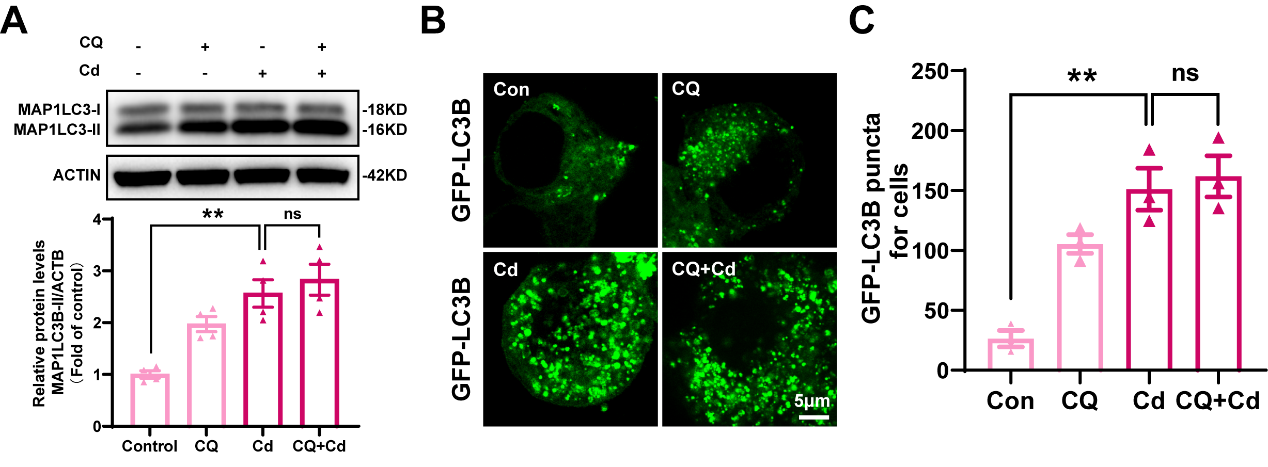


Figure S1. Cd exposure blocks autophagic flux in Neuro-2a cells. Representative immunoblot and quantification analysis of MAP1LC3B (A) and representative images and quantification of GFP-LC3B puncta (B, C) in Neuro-2a cells treated with or without CdCl_2_ (4 μM, 72 h) in the absence or presence of CQ (37.5 μM, 72 h). Scale bar: 5 μm. ***p* <0.01 vs. the control group. ns: not significant.


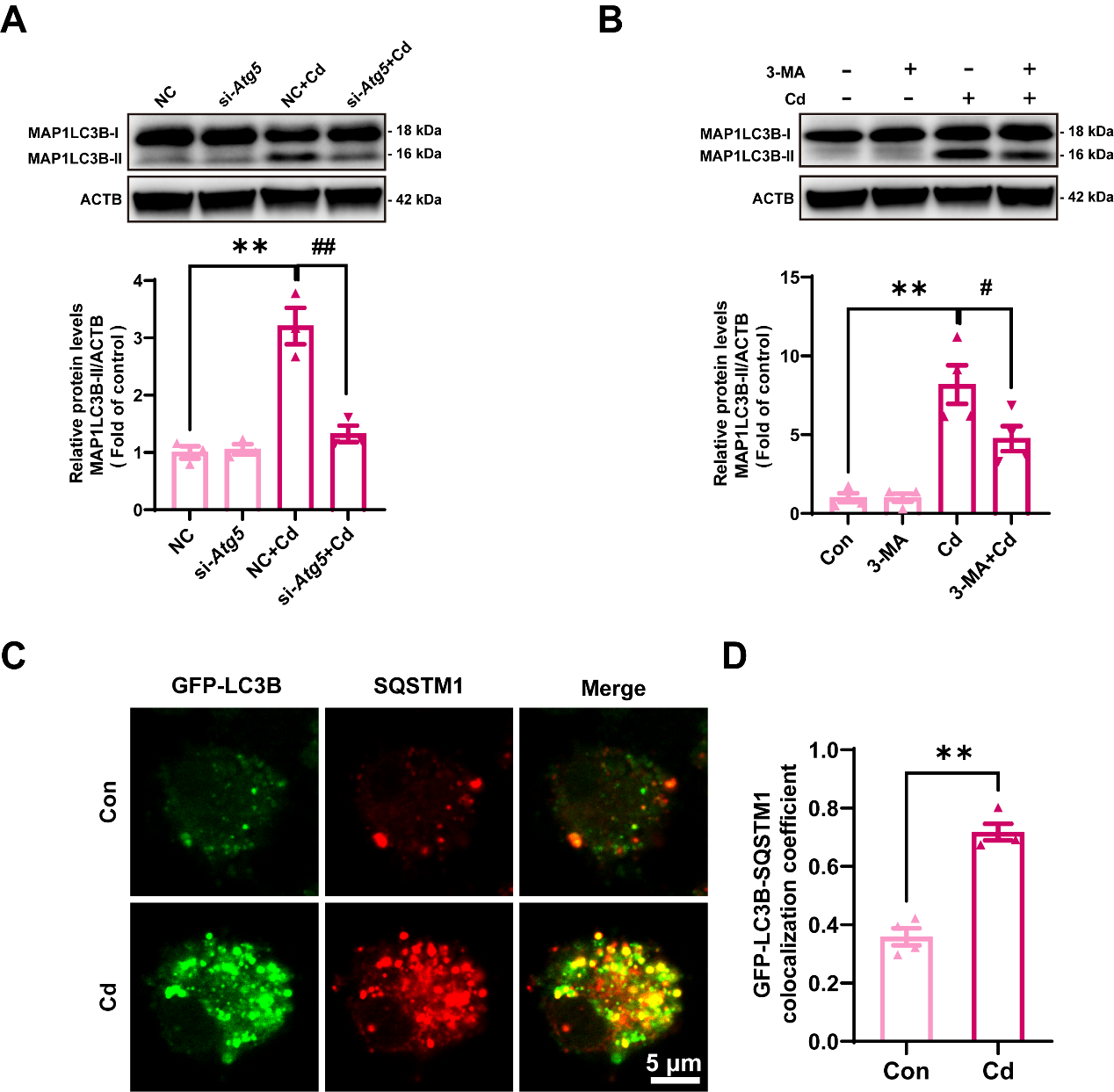
Figure S2. Cd exposure does not disturb phagophore formation or autophagosome maturation in Neuro-2a cells. (A) Representative immunoblot and quantification of MAP1LC3B in Neuro-2a cells transfected with NC or Atg5-siRNA (si-Atg5) and treated with or without CdCl2 (4 μM) for 72 h. (B) Representative immunoblot and quantification analysis of MAP1LC3B in Neuro-2a cells treated with or without Cd (4 μM) in the absence or presence of 3-MA (1 mM) for 72 h. (C, D) Representative images and colocalization coefficient analysis of GFP-LC3B and SQSTM1 in Neuro-2a cells treated with or without CdCl_2_ (4 μM) for 72 h. Scale bar: 5 μm. **p < 0.01 vs. the Con/NC group; ^#^p < 0.05 and ^##^p < 0.01 vs. the Cd-exposed groups.


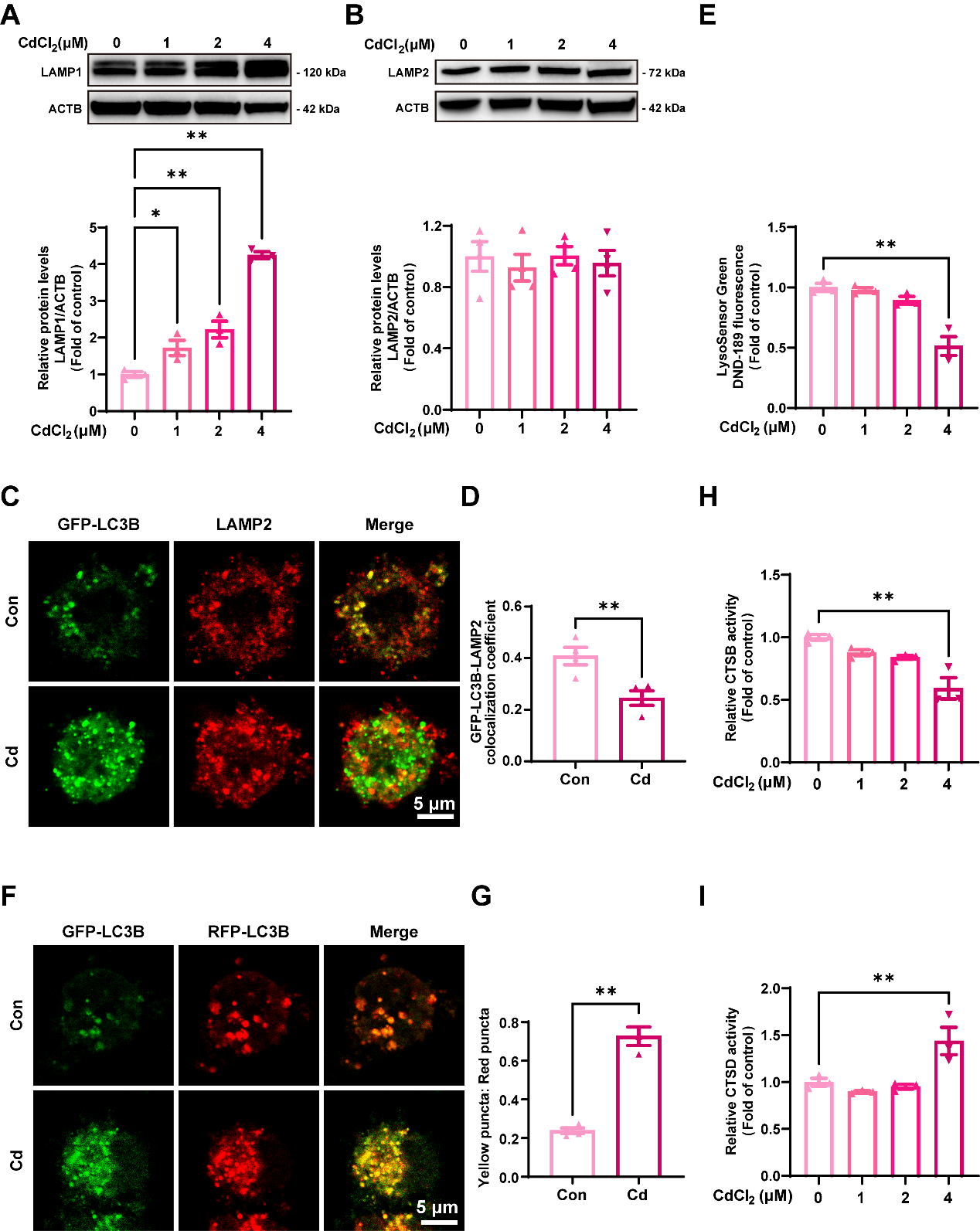


Figure S3. Cd exposure impairs autophagosome-lysosome fusion and lysosomal functions in Neuro-2a cells. Representative immunoblot and quantification analysis of LAMP1 (A) and LAMP2 (B) in Neuro-2a cells treated with Cd at different concentrations (0, 1, 2, or 4 μM) for 72 h. (C, D) Representative images and colocalization coefficient analysis of GFP-LC3B and LAMP2 in Neuro-2a cells treated with or without CdCl_2_ (4 μM) for 72 h. Scale bar: 5 μm. (E) LysoSensor DND-189 fluorescence intensity in Neuro-2a cells treated with Cd at different concentrations (0, 1, 2, or 4 μM) for 72 h. (F) Immunofluorescence analysis of Neuro-2a cells transfected with tandem sensor RFP-GFP-LC3B for 24 h and treated with Cd (4 μM) for an additional 72 h. The RFP-GFP-LC3B system is a tool used for fusion detection, in which the fluorescence of GFP is quenched by acidic environments. Pre-fusion autophagosomes are represented by yellow puncta, and post-fusion autophagosomes are represented by red puncta. Scale bar: 5 μm. (G) Ratio of yellow puncta to red puncta following the transfection of Neuro-2a cells with RFP-GFP-LC3B and treatment with Cd (4 μM) for 72 h. CTSB (H) and CTSD (I) activities in Neuro-2a cells treated with Cd at different concentrations (0, 1, 2, or 4 μM) for 72 h. **p* < 0.05 and ***p* < 0.01 vs. the Con groups.


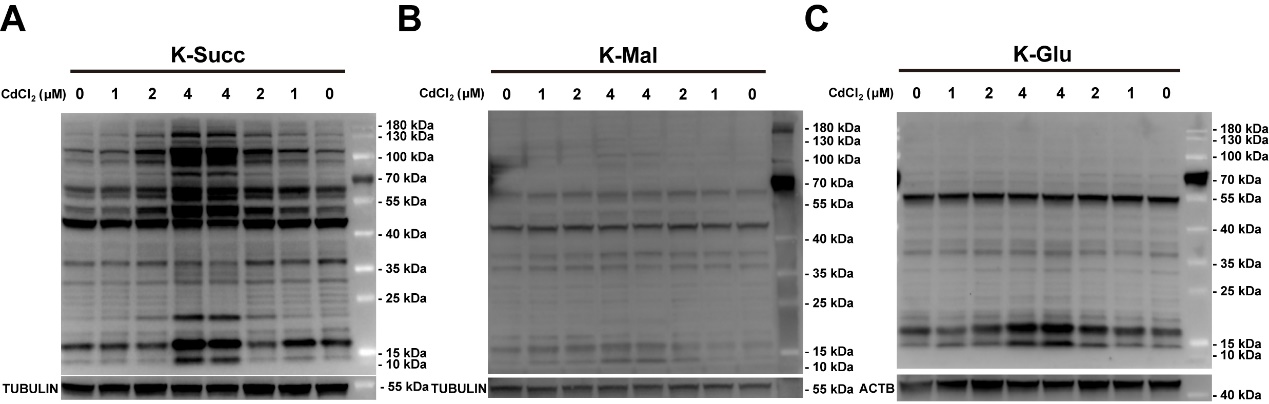


Figure S4. Cd exposure significantly increases the levels of lysine-succinylated proteins in Neuro-2a cells treated with Cd at different concentrations (0, 1, 2, or 4 μM) for 72 h. (A) Representative immunoblots of lysine-succinylated proteins (K-Succ) and TUBULIN obtained using a pan anti-succinyllysine antibody and an anti-TUBULIN antibody, respectively. (B) Representative immunoblots of lysine-malonylated proteins (K-Mal) and TUBULIN obtained using a pan anti-malonyllysine antibody and an anti-TUBULIN antibody, respectively. (C) Representative immunoblots of lysine-glutarylated proteins (K-Glu) and ACTB obtained using a pan anti-glutaryllysine antibody and an anti-ACTB antibody, respectively.


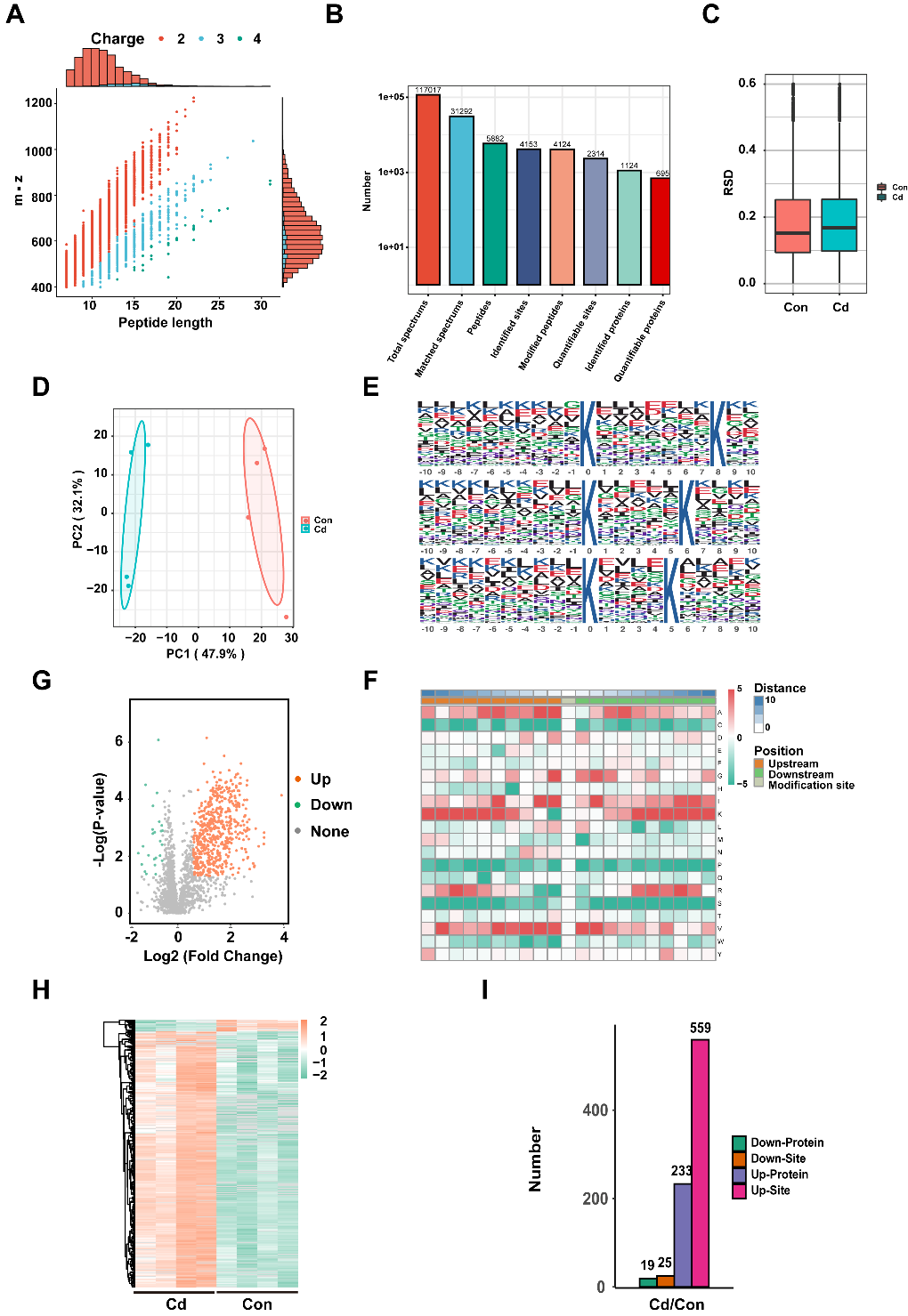


Figure S5. Quantitative global succinylome analysis of Neuro-2a cells treated with or without Cd for 72 h. (A, B) Basic statistical analysis of the MS data. (C, D) RSD and PCA of succinylation sites. (E) Motif analysis of the identified succinylation sites. (F) Motif enrichment heatmap of the amino acids upstream and downstream of the identiﬁed modiﬁcation sites. Volcano plot (G) and clustering heatmap (H) of the differentially expressed succinylated modiﬁcation sites (orange: increased; green: decreased). (I) Number of sites with different levels of succinylation modiﬁcation and proteins with differentially expressed succinylation sites in Cd-exposed Neuro-2a cells compared with those in control cells.


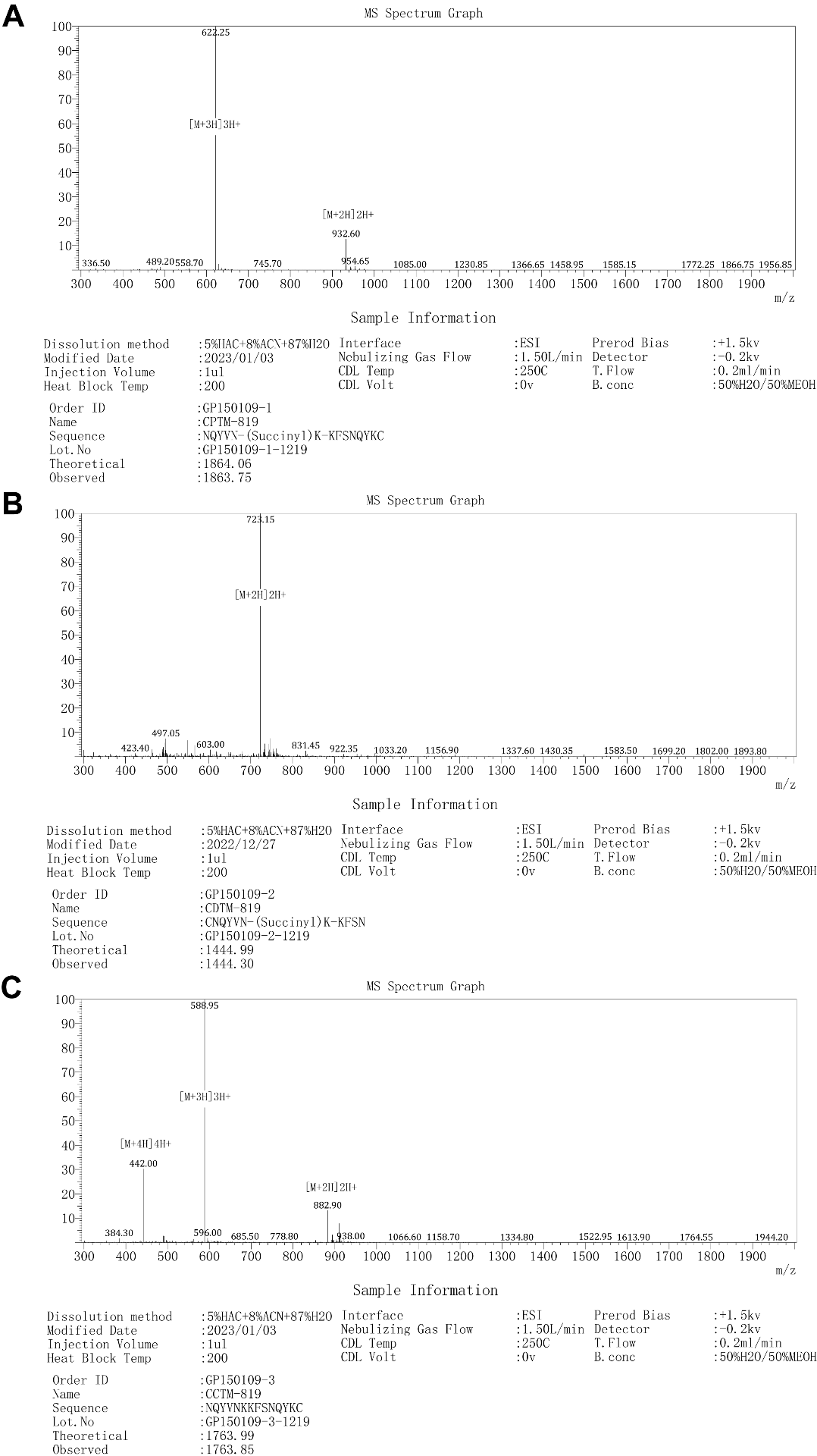


Figure S6. The results of MS detection. (A) RAB7A K31^su^ Peptide 1. (B) RAB7A K31^su^ Peptide 2. (C) Non-modified control peptide.


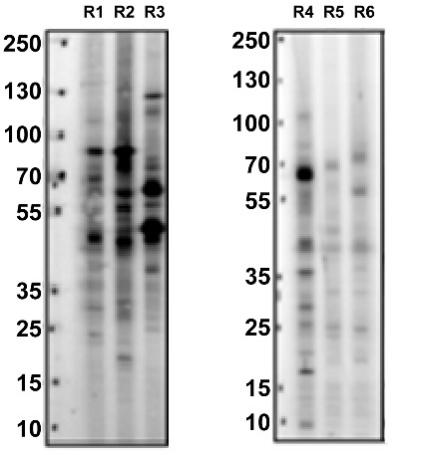


Figure S7. The detection of Western blot in HeLa cells using the rabbit serums.


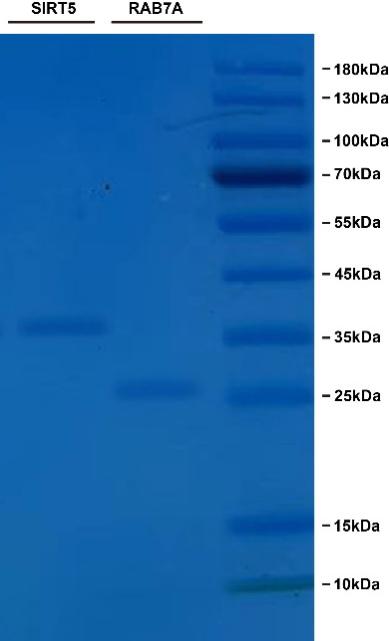


Figure S8. Purified recombinant mouse proteins are separated via sodium dodecyl sulfate‐polyacrylamide gel electrophoresis (SDS‐PAGE) on a 12% agarose gel and stained with Coomassie Brilliant Blue.


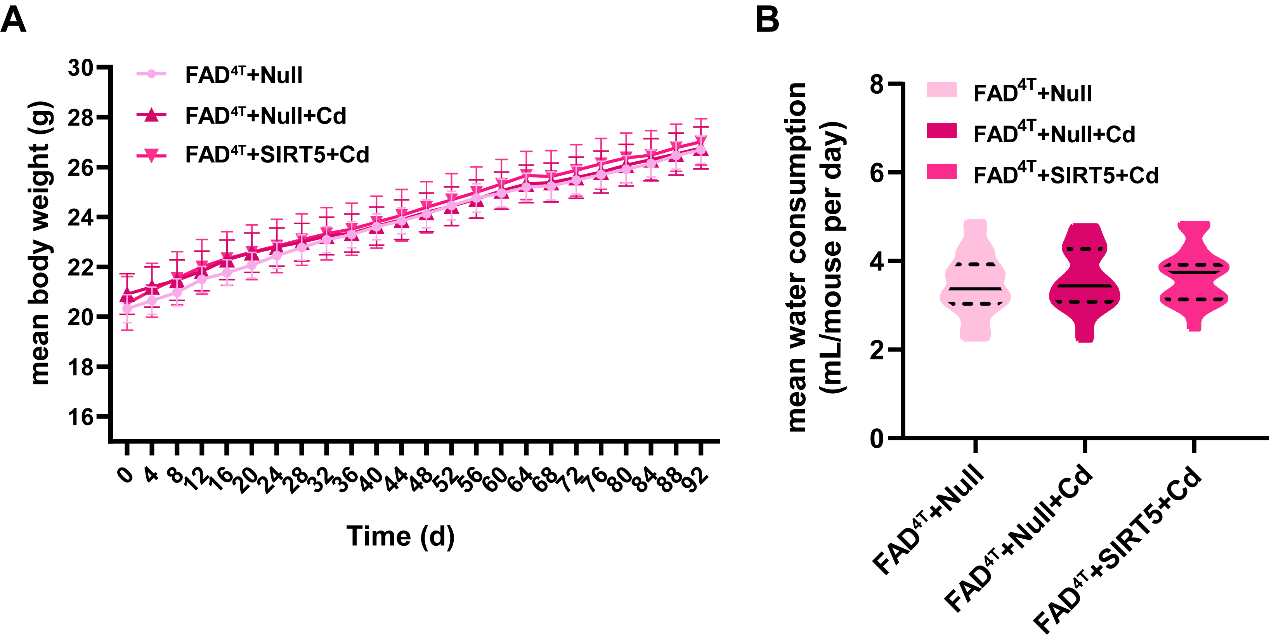


Figure S9. The mean body weight and water consumption dose not significantly differ among the groups. All FAD^4T^ mice were intravenously injected with AAV-*Null* or AAV-*Sirt5* and exposed or not exposed to 3.6 mg/L CdCl_2_ for 12 weeks. (A) Mean body weight, n = 12 mice/group. (B) Mean daily water consumption by each mouse. n = 12 mice/group.


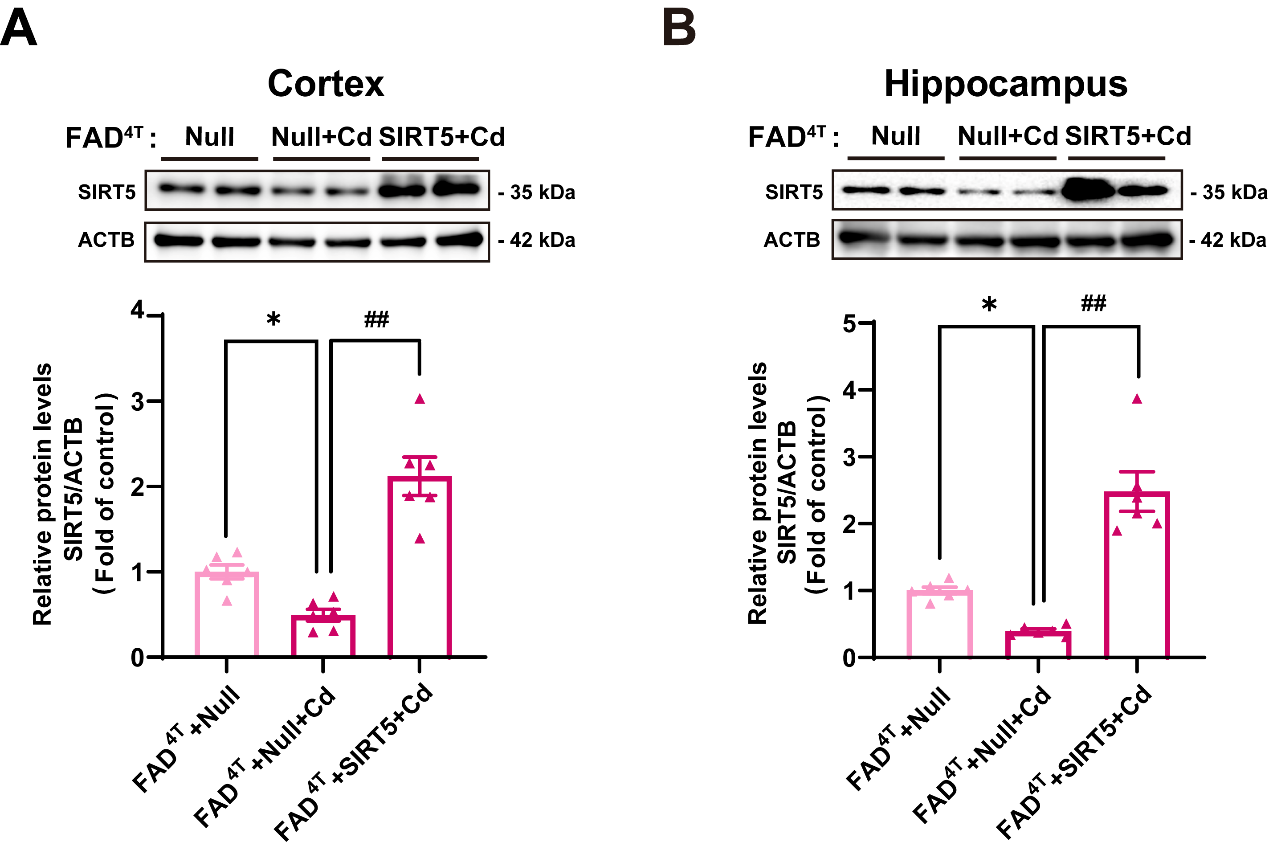


Figure S10. SIRT5 overexpression antagonizes Cd-induced inhibition of SIRT5 expression *in vivo*. All FAD^4T^ mice were intravenously injected with AAV-*Null* or AAV-*Sirt5* and exposed or not exposed to 3.6 mg/L CdCl_2_ for 12 weeks. (A) SIRT5 levels in the cortex of FAD^4T^ mice. n = 6 mice/group. (B) SIRT5 levels in the hippocampus of FAD^4T^ mice. n = 6 mice/group. **p* <0.05 vs. the FAD^4T^+Null group; ^##^*p* < 0.01 vs. the FAD^4T^+Null+Cd group.


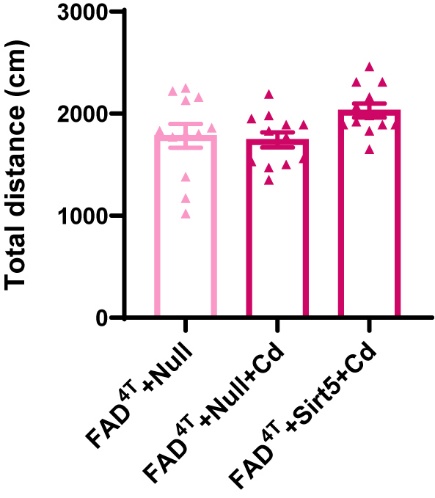


Figure S11. SIRT5 overexpression did not perturb the locomotor activities of FAD^4T^ mice. All the FAD^4T^ mice were intravenously injected with AAV-*Null* or AAV-*Sirt5* and exposed or not exposed to 3.6 mg/L CdCl_2_ for 12 weeks. The total distance traveled by FAD^4T^ mice was consistent across all groups in the Y-maze test. n = 12 mice/group.


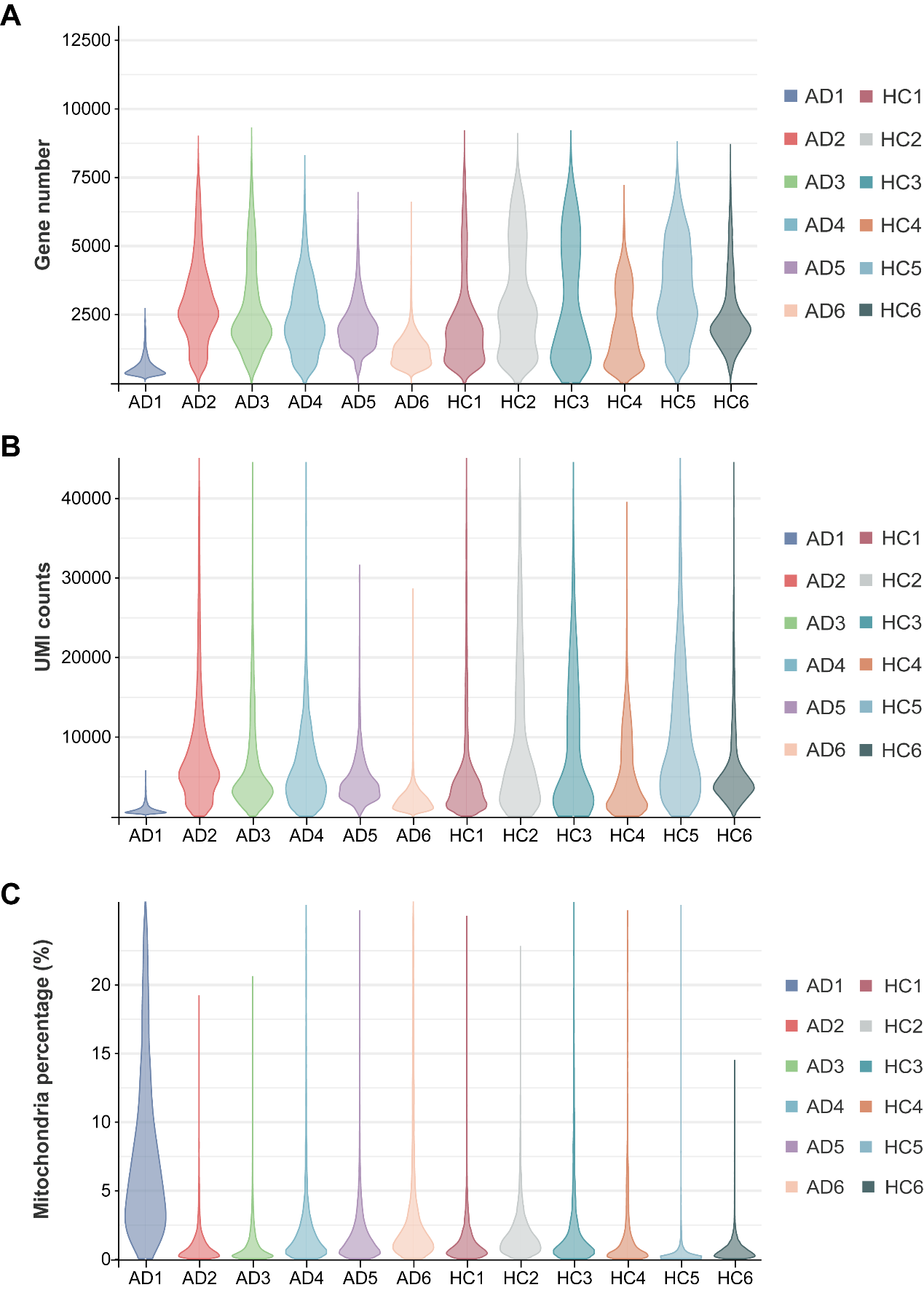


Figure S12. snRNA-Seq data processing. The data processing of all samples including AD patients and healthy controls (HC) in gene number (A), UMI counts (B) and mitochondria percentage (C).


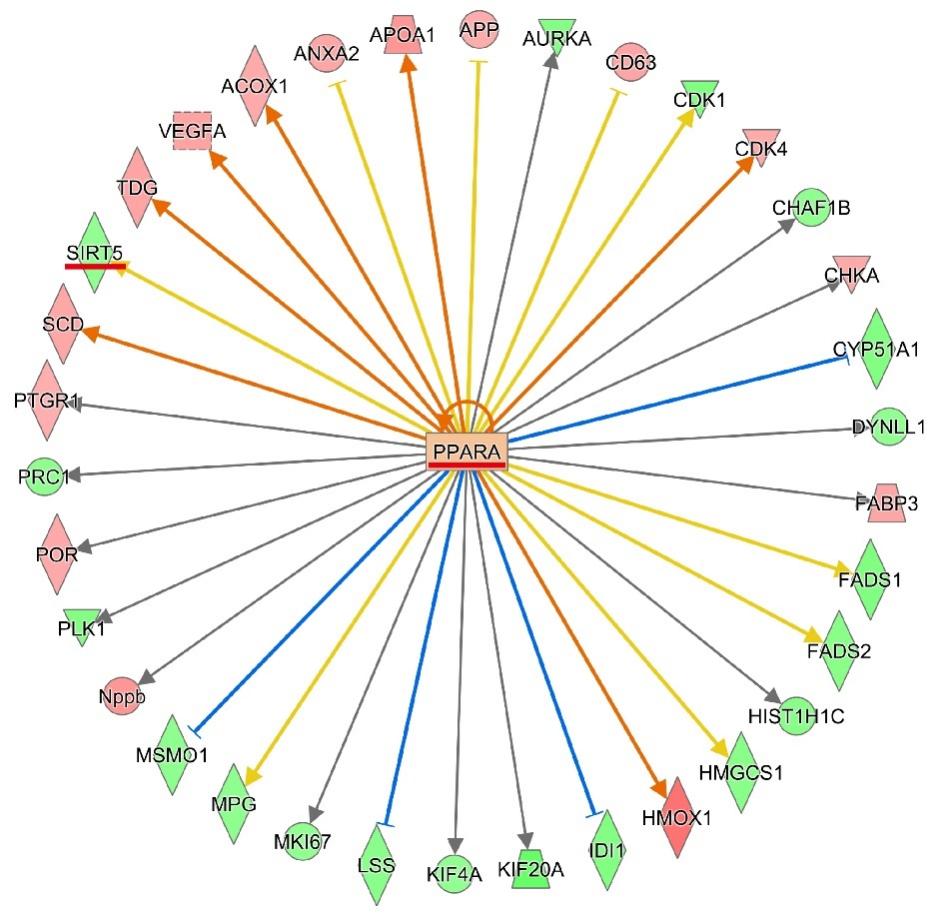


Figure S13. The result of upstream regulator analysis was performed by the IPA algorithm based on the differentially expressed proteins in Cd-exposed Neuro-2a cells compared with control cells.

**Table S1. Polypeptide information sheet**

| Name | Sequences | Modified type | Weight |
| --- | --- | --- | --- |
| Peptide 1 | NQYVN-(Succinyl)K-KFSNQYK C | succinyl-modified peptide | 1863.75 |
| Peptide 2 | CNQYVN-(Succinyl)K-KFSN | succinyl-modified peptide | 1444.30 |
| Peptide 3 | NQYVNKKFSNQYKC | Non-modified control peptide | 1763.85 |

**Table S2-1. The ELISA results of rabbit serum**

**(RAB7A K31^su^ Peptide 1)**

| **Dilute concentration** | **Rabbit** | | | | | |
| --- | --- | --- | --- | --- | --- | --- |
|  | **R1** | **R2** | **R3** | **R4** | **R5** | **R6** |
| **1:250** | 1.949 | 1.889 | 1.921 | 1.572 | 1.789 | 1.665 |
| **1:1K** | 1.548 | 1.651 | 1.475 | 1.021 | 1.668 | 1.459 |
| **1:4K** | 1.011 | 1.458 | 1.126 | 0.571 | 1.132 | 1.128 |
| **1:16K** | 0.449 | 0.867 | 0.568 | 0.204 | 0.572 | 0.478 |
| **1:64K** | 0.143 | 0.323 | 0.188 | 0.084 | 0.218 | 0.164 |
| **1:256K** | 0.071 | 0.144 | 0.135 | 0.06 | 0.089 | 0.077 |
| **1:1024k** | 0.047 | 0.072 | 0.052 | 0.053 | 0.052 | 0.056 |
| **1:4096k** | 0.048 | 0.055 | 0.052 | 0.053 | 0.069 | 0.044 |

**Table S2-2. The ELISA results of rabbit serum**

**(RAB7A K31^su^ Peptide 2)**

| **Dilute concentration** | **Rabbit** | | | | | |
| --- | --- | --- | --- | --- | --- | --- |
|  | **R1** | **R2** | **R3** | **R4** | **R5** | **R6** |
| **1:250** | 1.636 | 1.855 | 1.361 | 1.73 | 1.753 | 1.515 |
| **1:1K** | 1.236 | 1.597 | 0.878 | 1.587 | 1.667 | 1.645 |
| **1:4K** | 0.634 | 1.22 | 0.398 | 1.108 | 1.331 | 1.512 |
| **1:16K** | 0.26 | 0.648 | 0.153 | 0.546 | 0.858 | 1.056 |
| **1:64K** | 0.084 | 0.224 | 0.068 | 0.18 | 0.339 | 0.514 |
| **1:256K** | 0.348 | 0.1 | 0.054 | 0.087 | 0.121 | 0.162 |
| **1:1024k** | 0.059 | 0.061 | 0.058 | 0.056 | 0.063 | 0.102 |
| **1:4096k** | 0.052 | 0.059 | 0.054 | 0.046 | 0.047 | 0.063 |

**Table S2-3. The ELISA results of rabbit serum**

**(Non-modified control peptide)**

| **Dilute concentration** | **Rabbit** | | | | | |
| --- | --- | --- | --- | --- | --- | --- |
|  | **R1** | **R2** | **R3** | **R4** | **R5** | **R6** |
| **1:250** | 1.719 | 1.439 | 1.497 | 0.183 | 0.542 | 0.143 |
| **1:1K** | 1.341 | 1.427 | 1.381 | 0.075 | 0.177 | 0.067 |
| **1:4K** | 0.778 | 0.785 | 0.822 | 0.044 | 0.075 | 0.047 |
| **1:16K** | 0.301 | 0.373 | 0.405 | 0.042 | 0.053 | 0.05 |
| **1:64K** | 0.122 | 0.129 | 0.15 | 0.041 | 0.047 | 0.044 |
| **1:256K** | 0.073 | 0.07 | 0.076 | 0.041 | 0.046 | 0.057 |
| **1:1024k** | 0.061 | 0.056 | 0.051 | 0.04 | 0.042 | 0.041 |
| **1:4096k** | 0.048 | 0.043 | 0.043 | 0.043 | 0.046 | 0.044 |

**Table S3. The ELISA results of rabbit anti-RAB7A-succinyl-K31 antibody detection**

| **Dilute concentration** | **Ab4** | | | **Ab5** | | | **Ab6** | | |
| --- | --- | --- | --- | --- | --- | --- | --- | --- | --- |
|  | **K31^su^ Peptide 1** | **K31^su^ Peptide 2** | **Control peptide** | **K31^su^ Peptide 1** | **K31^su^ Peptide 2** | **Control peptide** | **K31^su^ Peptide 1** | **K31^su^ Peptide 2** | **Control peptide** |
| **1:2K** | 1.285 | 1.669 | 0.534 | 3.355 | 3.221 | 0.144 | 1.876 | 1.363 | 0.089 |
| **1:6K** | 1.207 | 1.468 | 0.239 | 3.247 | 2.98 | 0.078 | 1.457 | 1.253 | 0.065 |
| **1:18K** | 1.124 | 1.448 | 0.099 | 2.883 | 2.942 | 0.071 | 1.35 | 1.102 | 0.043 |
| **1:54K** | 1.04 | 1.322 | 0.065 | 1.98 | 2.521 | 0.059 | 0.872 | 0.791 | 0.06 |
| **1:162K** | 0.599 | 0.816 | 0.048 | 0.982 | 1.455 | 0.048 | 0.427 | 0.64 | 0.046 |
| **1:486K** | 0.248 | 0.395 | 0.045 | 0.464 | 0.652 | 0.063 | 0.202 | 0.271 | 0.061 |
| **1:1458k** | 0.121 | 0.173 | 0.056 | 0.213 | 0.294 | 0.063 | 0.107 | 0.143 | 0.066 |
| **1:4374k** | 0.072 | 0.1 | 0.056 | 0.13 | 0.161 | 0.078 | 0.079 | 0.101 | 0.054 |

**Table S4. Hydrogen bonding sites identified by molecular docking**

| Receptor  (A) | Ligand  (B) | Hydrogen Bond Interaction (2.5Å) |
| --- | --- | --- |
| RAB7A  (5Z2M) | SIRT5  (Alphafold predicted) | A:31:LYS-B:255:TYR  A:6:LYS-B:283:ASP |
|  |  | A:27:GLN-B:226:ASN |
|  |  | A:159:ALA-B:89:GLN |

**Table S5. Detailed information for sn-RNA seq analysis**

| **Dataset** | **Serial number** | **Raw data information** | **Sample sign** | **Group information** |
| --- | --- | --- | --- | --- |
| GSE188545 | GSM5685287 | AD02MTG | AD1 | AD patients |
|  | GSM5685288 | AD12MTG | AD2 |  |
|  | GSM5685289 | AD30MTG | AD3 |  |
|  | GSM5685290 | AD04MTG | AD4 |  |
|  | GSM5685291 | AD16MTG | AD5 |  |
|  | GSM5685292 | AD17MTG | AD6 |  |
|  | GSM5685293 | HC14MTG | HC1 | Health control (HC) |
|  | GSM5685294 | HC19MTG | HC2 |  |
|  | GSM5685295 | HC35MTG | HC3 |  |
|  | GSM5685296 | HC03MTG | HC4 |  |
|  | GSM5685297 | HC07MTG | HC5 |  |
|  | GSM5685298 | HC37MTG | HC6 |  |

**Table S6. Antibodies used in this study**

| Antigen | Catalogue number | Supplier | Dilution ratio | Application |
| --- | --- | --- | --- | --- |
| APP | DF6012 | Affinity | 1:1000 | Western blot |
| MAP1LC3B | L7543 | SIGMA | 1:1000 | Western blot |
|  |  |  | 1:200 | Immunohistochemistry  (Brain slides) |
| SQSTM1 | Ab56416 | Abcam | 1:200 | Immunohistochemistry |
|  | GB11239-1-100 | Servicebio | 1:200 | Immunohistochemistry (Brain slides) |
| LAMP1 | Ab24170 | Abcam | 1:1000 | Western blot |
| LAMP2 | PA1-655 | Invitrogen | 1:1000 | Western blot |
|  | Ab13524 | Abcam | 1:100 | Immunohistochemistry |
| SIRT5 | DF8294 | Affinity | 1:1000 | Western blot |
|  | DF8294 | Affinity | 1:100 | Immunohistochemistry |
| Succinyllysine | PTM-419 | PTM BIO | 1:500 | Western blot |
| Malonyllysine | PTM-902 | PTM BIO | 1:500 | Western blot |
| Glutaryllysine | PTM-1151 | PTM BIO | 1:500 | Western blot |
| RAB7A K31^su^ | CM1118 | PTM BIO | 1:500 | Western blot |
|  |  |  | 1:2000 | Dot blot |
| RAB7A | Ab50533 | Abcam | 1:100 | Immunohistochemistry |
| RILP | 13574-1-AP | Proteintech | 1:100 | Immunohistochemistry |
| ATP6V0D1 | BS5977 | Bioworld | 1:1000 | Western blot |
| ATP6V1E1 | BS72008 | Bioworld | 1:1000 | Western blot |
| Beta-Amyloid | Ab11132 | Abcam | 1:200 | Immunohistochemistry  (Brain slides) |
| ACTB | A1978 | SIGMA | 1:5000 | Western blot |
| TUBULIN | T2200 | SIGMA | 1:5000 | Western blot |
| Goat Anti-Mouse IgG (HRP) | A0216 | 碧云天 | 1:1000 | Western blot |
| Goat Anti-Rabbit IgG (HRP) | Ab205718 | Abcam | 1:2000 | Western blot |
|  |  |  | 1:10000 | Dot blot |
| Alexa Fluor™ 568, Donkey anti-Mouse IgG | A10037 | Invitrogen | 1:200 | Immunohistochemistry |
| Alexa Fluor™ 488, Donkey anti-Rabbit IgG | A21206 | Invitrogen | 1:200 | Immunohistochemistry |
| Alexa Fluor™ 568, Donkey anti-Rat IgG | A78946 | Invitrogen | 1:200 | Immunohistochemistry |
| Alexa Fluor™ 488, Goat anti-Rabbit IgG | GB25303 | Servicebio | 1:200 | Immunohistochemistry  (Brain slides) |
| Goat Anti-Mouse IgG (HRP) | GB23301 | Servicebio | 1:200 | Immunohistochemistry  (Brain slides) |

All immunofluorescence images with a complete field of view

Figure 1H


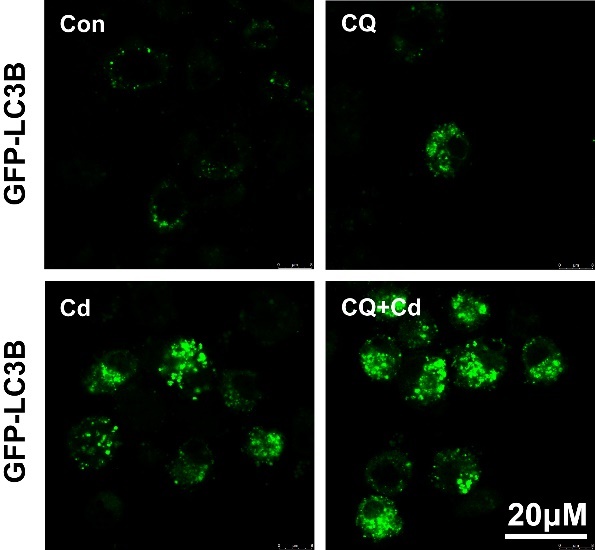


Figure 3B


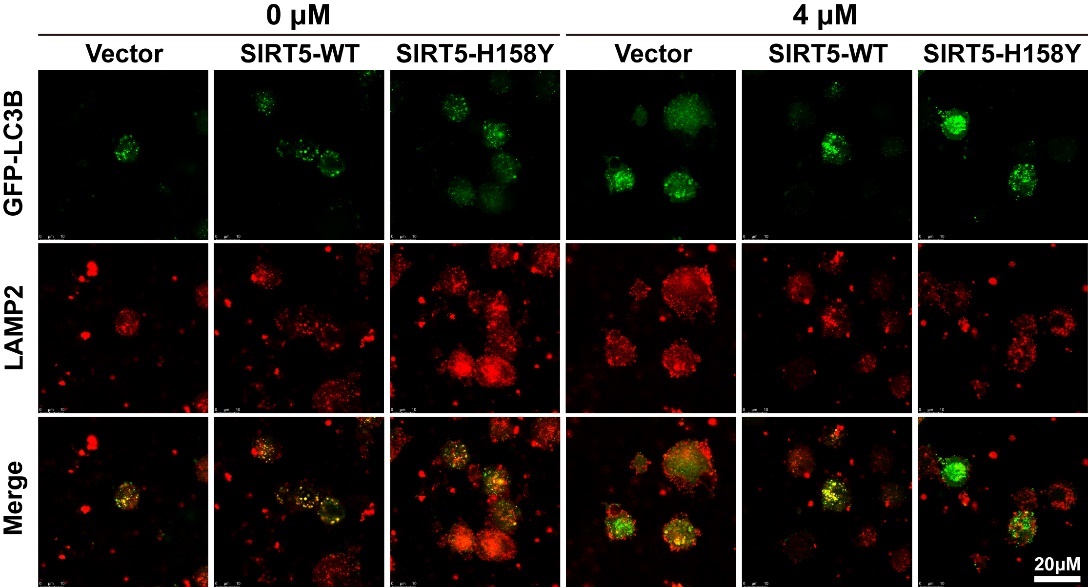


Figure 3D


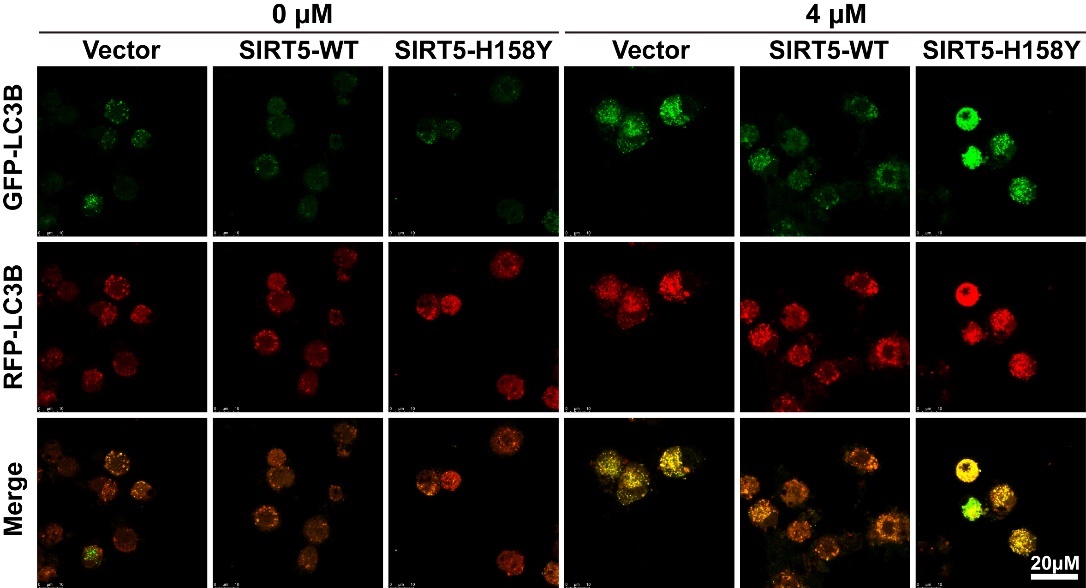


Figure 5A


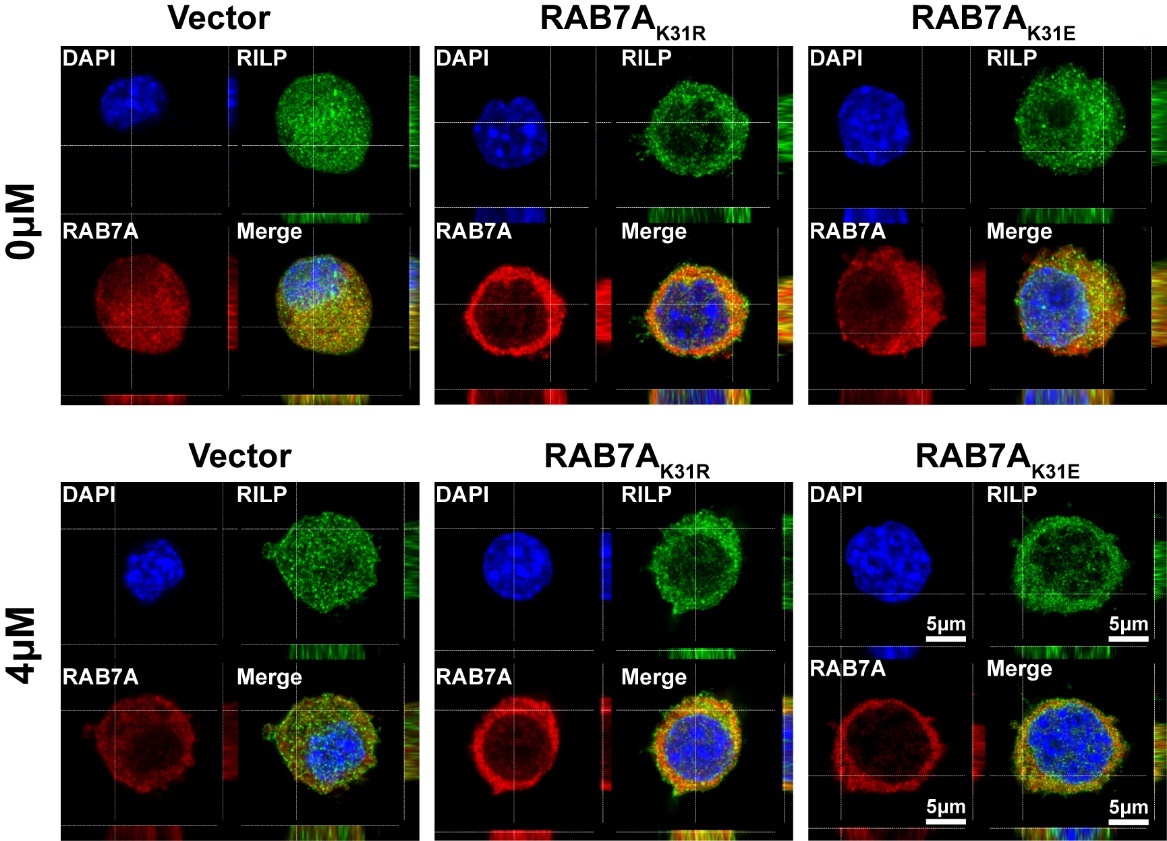


Figure 5C


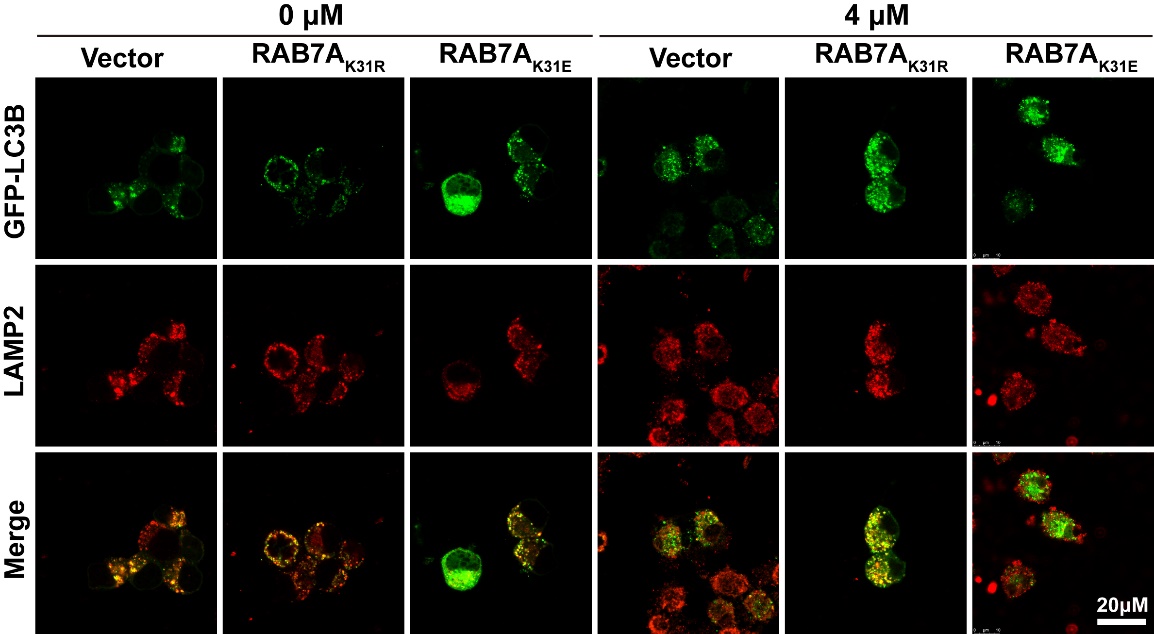


Figure 5E


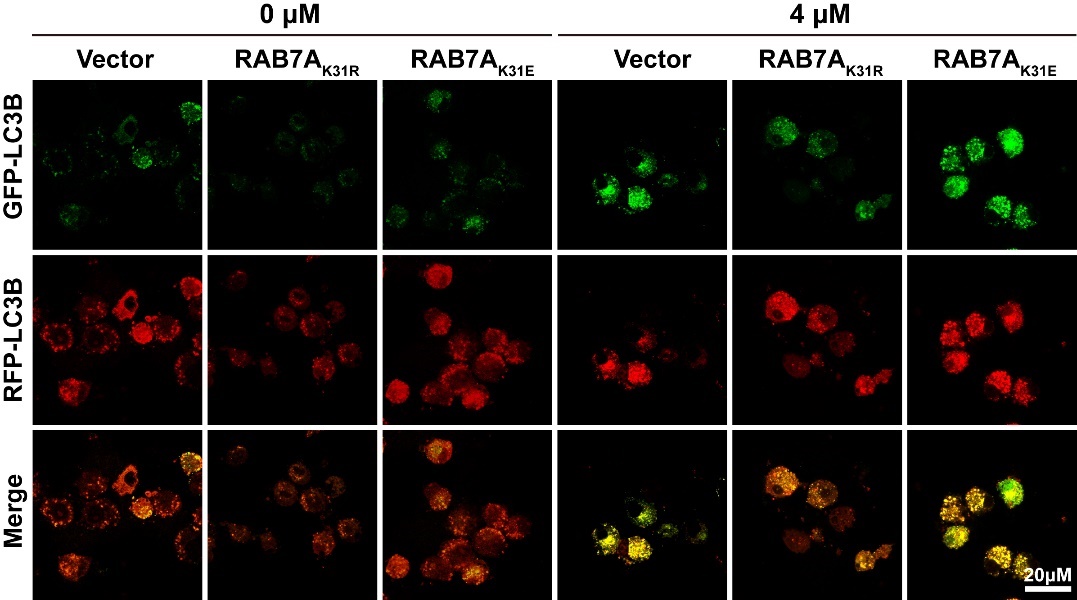


Figure 6D


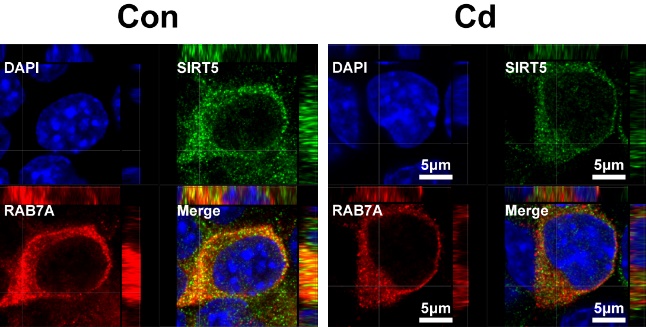


Figure 6G


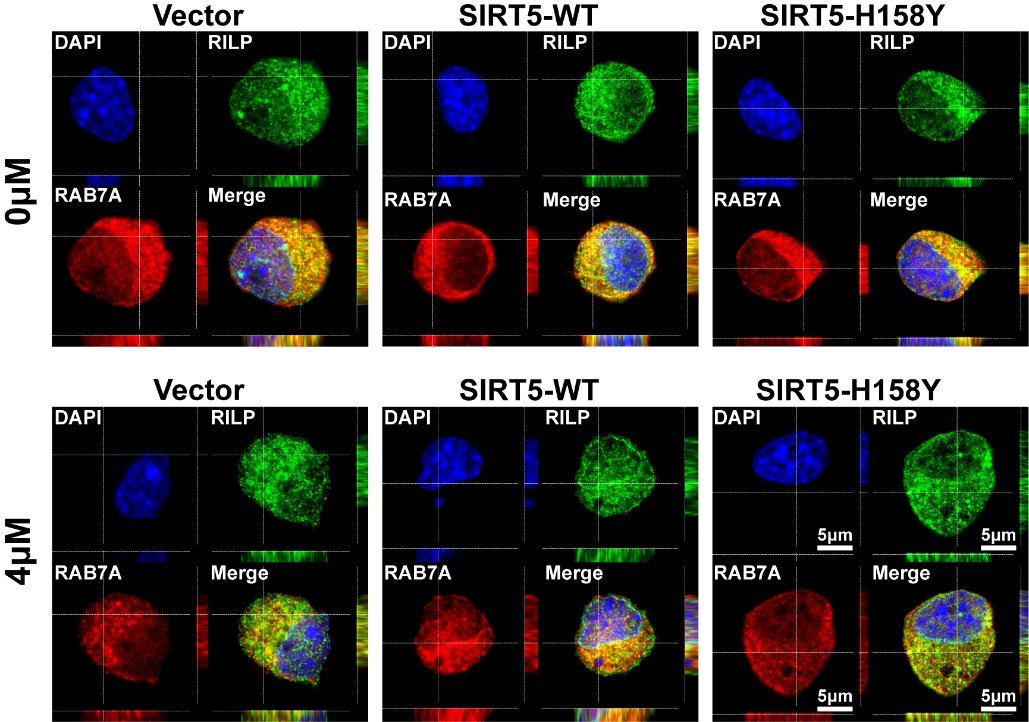


Figure S1B


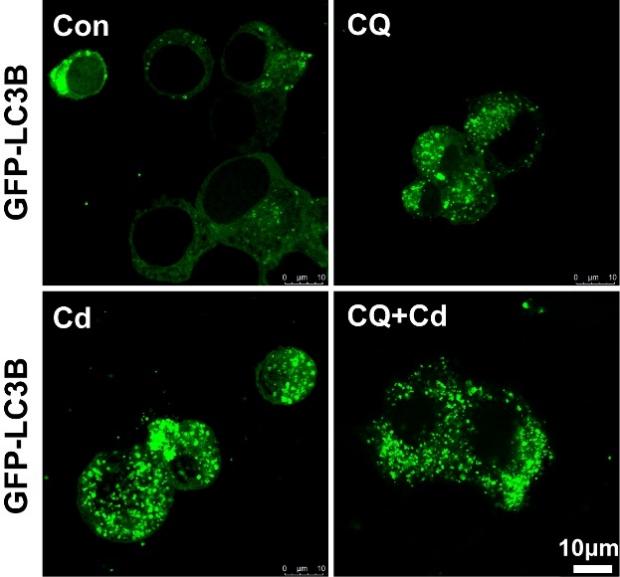


Figure S2C


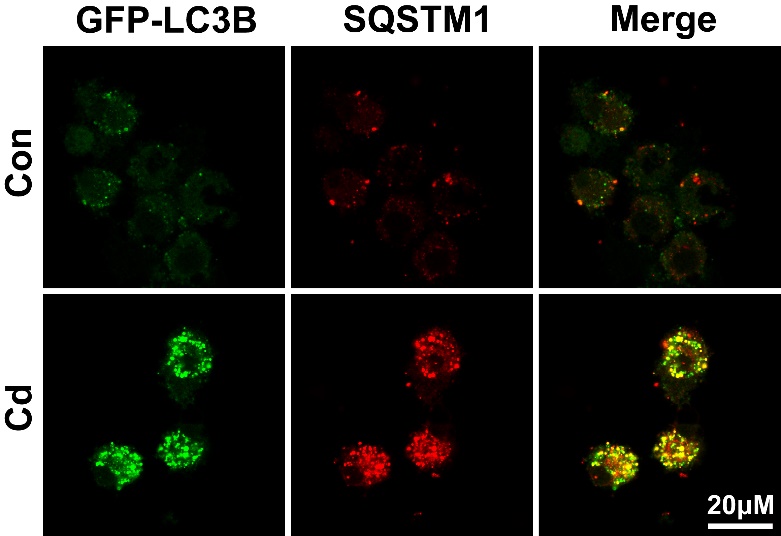


Figure S3C


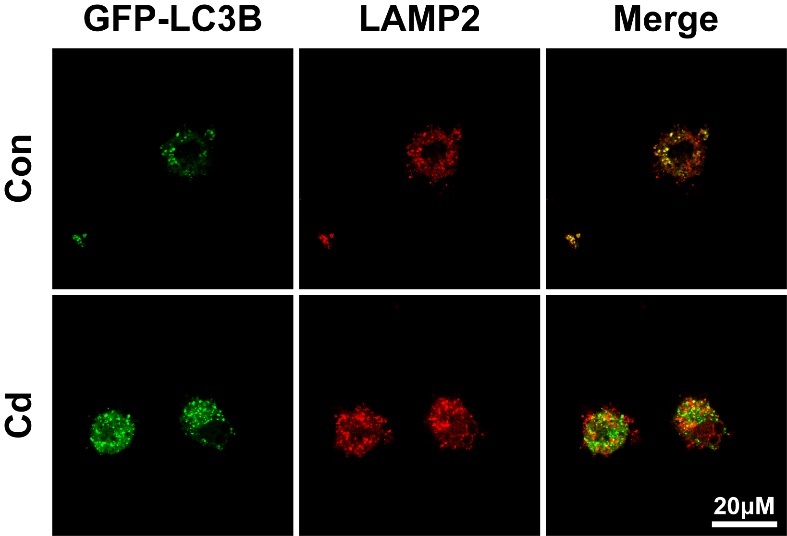


Figure S3F


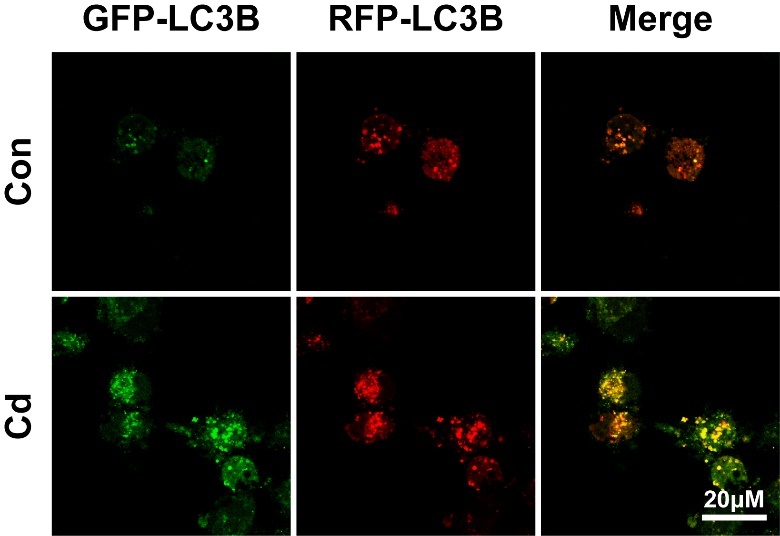


All full western blot data in the manuscript

Figure 1C


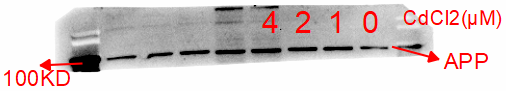


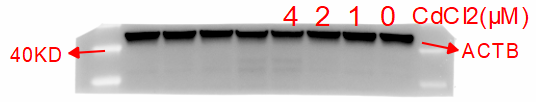


Figure 1F


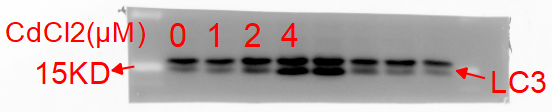


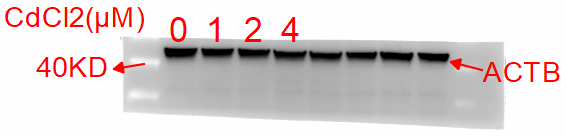


Figure 1G


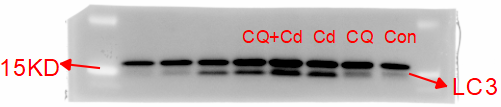


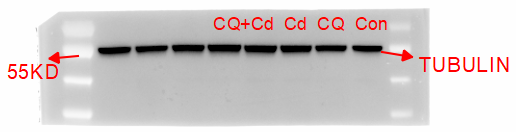


Figure 2C


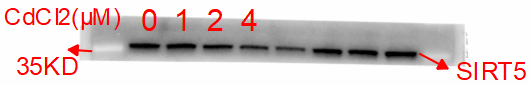


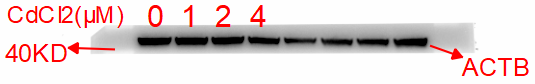


Figure 2D


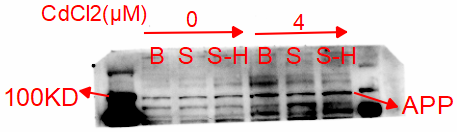


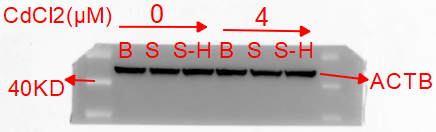


B: Vector S: SIRT5-WT S-H: SIRT5-H158Y

Figure 3A


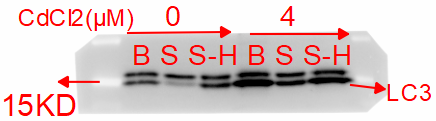


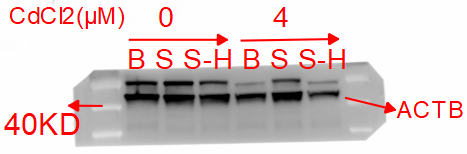


B: Vector S: SIRT5-WT S-H: SIRT5-H158Y

Figure 4G


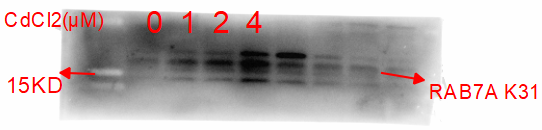


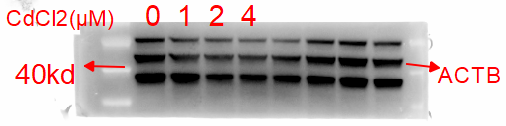


RAB7A K31: RAB7A K31^su^

Figure 5I


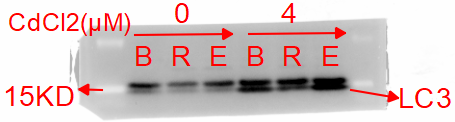


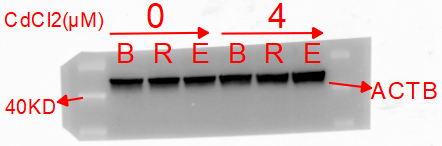


B: Vector R: RAB7A_K31R_  E: RAB7A_K31E_

Figure 5J


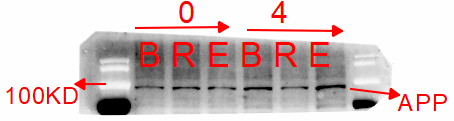


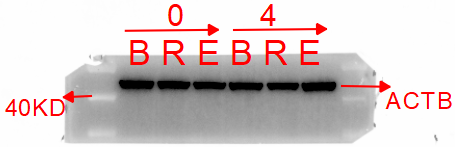


B: Vector R: RAB7A_K31R_  E: RAB7A_K31E_

Figure 6F


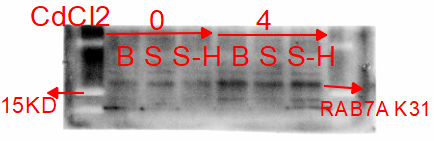


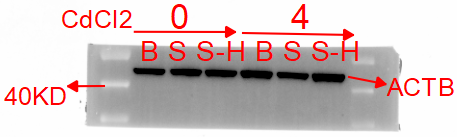


B: Vector S: SIRT5-WT S-H: SIRT5-H158Y

RAB7A K31: RAB7A K31^su^

Figure 8D


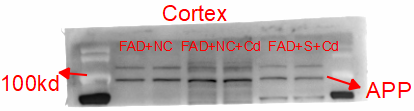


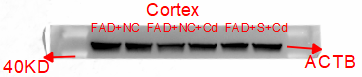


FAD+NC: FAD^4T^+Null FAD+NC+Cd: FAD^4T^+Null+Cd

FAD+S+Cd: FAD^4T^+SIRT5+Cd

Figure 8E


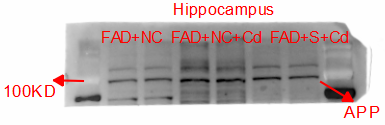


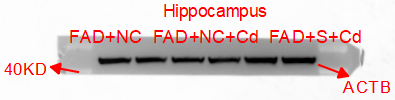


FAD+NC: FAD^4T^+Null FAD+NC+Cd: FAD^4T^+Null+Cd

FAD+S+Cd: FAD^4T^+SIRT5+Cd

Figure 8F


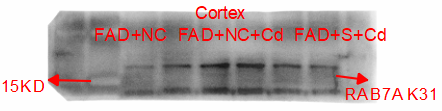


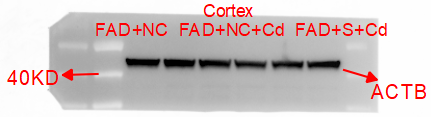


FAD+NC: FAD^4T^+Null FAD+NC+Cd: FAD^4T^+Null+Cd

FAD+S+Cd: FAD^4T^+SIRT5+Cd; RAB7A K31: RAB7A K31^su^

Figure 8G


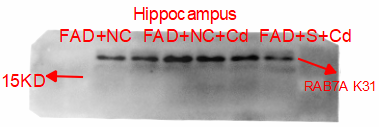


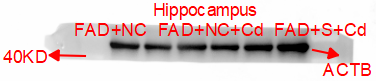


FAD+NC: FAD^4T^+Null FAD+NC+Cd: FAD^4T^+Null+Cd

FAD+S+Cd: FAD^4T^+SIRT5+Cd; RAB7A K31: RAB7A K31^su^

Figure 9G


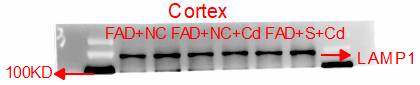


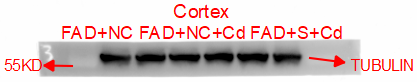


FAD+NC: FAD^4T^+Null FAD+NC+Cd: FAD^4T^+Null+Cd

FAD+S+Cd: FAD^4T^+SIRT5+Cd

Figure 9H


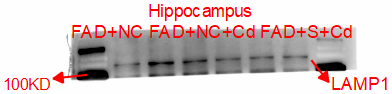


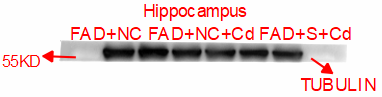


FAD+NC: FAD^4T^+Null FAD+NC+Cd: FAD^4T^+Null+Cd

FAD+S+Cd: FAD^4T^+SIRT5+Cd

Figure 9I


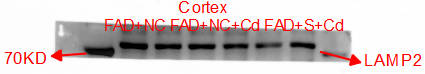


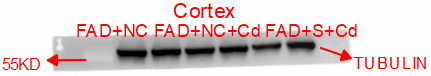


FAD+NC: FAD^4T^+Null FAD+NC+Cd: FAD^4T^+Null+Cd

FAD+S+Cd: FAD^4T^+SIRT5+Cd

Figure 9J


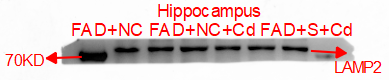


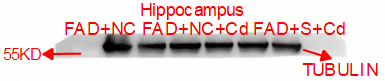


FAD+NC: FAD^4T^+Null FAD+NC+Cd: FAD^4T^+Null+Cd

FAD+S+Cd: FAD^4T^+SIRT5+Cd

Figure 9K


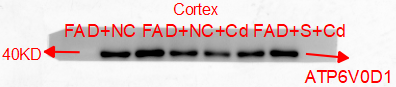


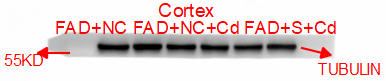


FAD+NC: FAD^4T^+Null FAD+NC+Cd: FAD^4T^+Null+Cd

FAD+S+Cd: FAD^4T^+SIRT5+Cd

Figure 9L


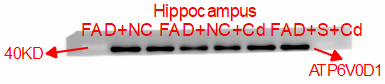


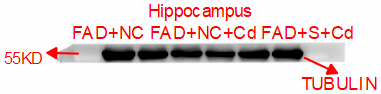


FAD+NC: FAD^4T^+Null FAD+NC+Cd: FAD^4T^+Null+Cd

FAD+S+Cd: FAD^4T^+SIRT5+Cd

Figure 9M


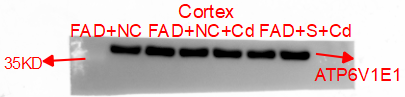


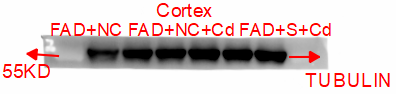


FAD+NC: FAD^4T^+Null FAD+NC+Cd: FAD^4T^+Null+Cd

FAD+S+Cd: FAD^4T^+SIRT5+Cd

Figure 9N


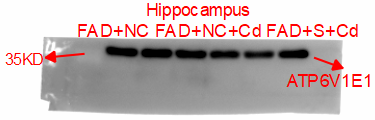


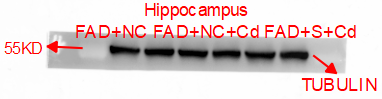


FAD+NC: FAD^4T^+Null FAD+NC+Cd: FAD^4T^+Null+Cd

FAD+S+Cd: FAD^4T^+SIRT5+Cd

Figure S1A


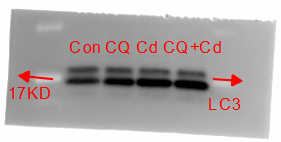


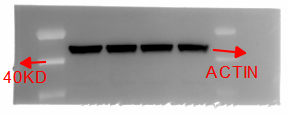


Figure S2A


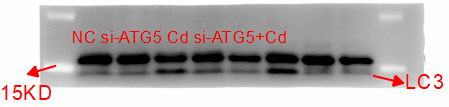


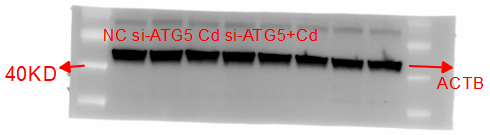


Figure S2B


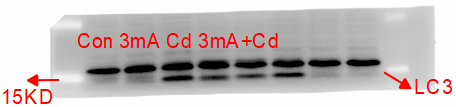


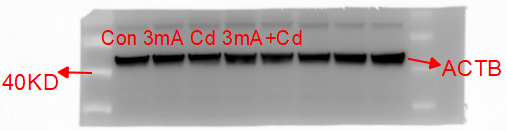


Figure S3A


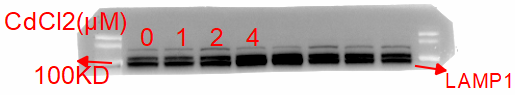


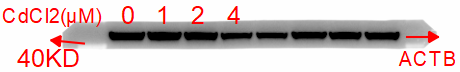


Figure S3B


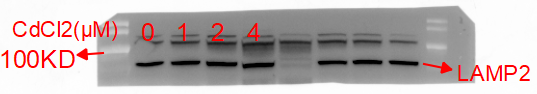


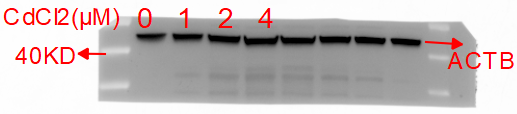


Figure S4A


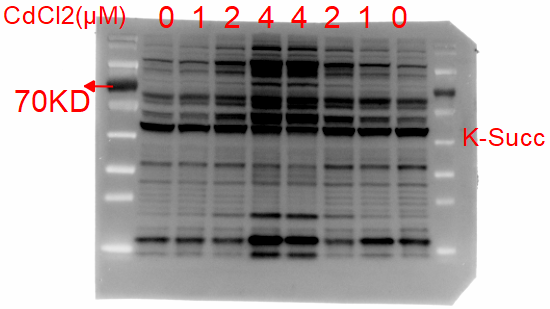

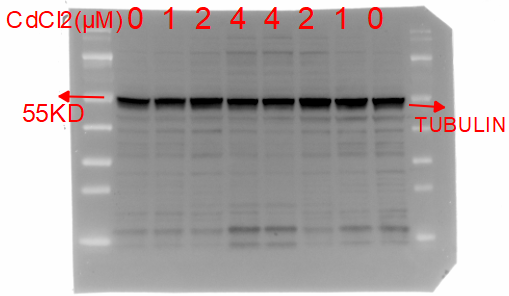


K-Succ: Succinyllysine

Figure S4B


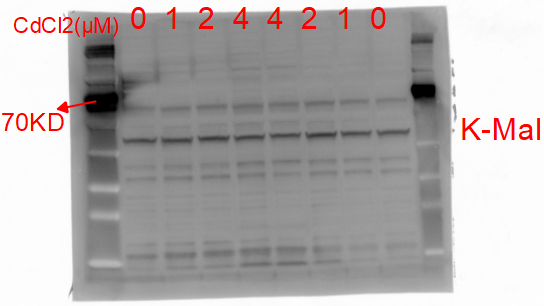

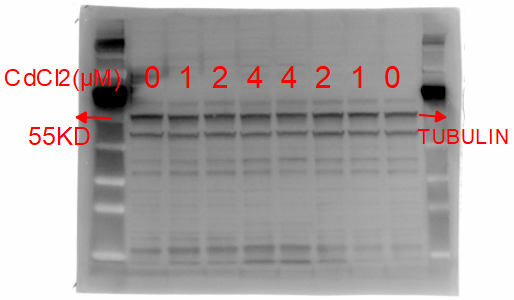


K-Mal: Malonyllysine

Figure S4C


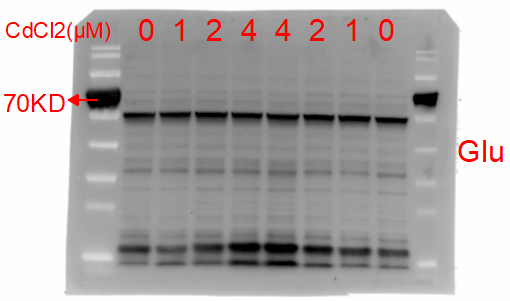

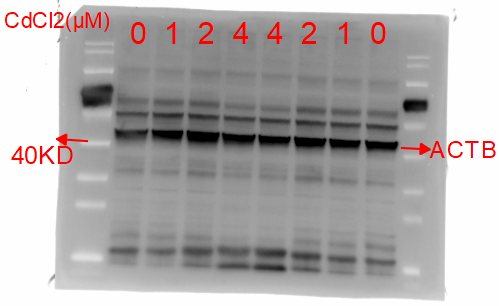


Glu: Glutaryllysin

Figure S10A


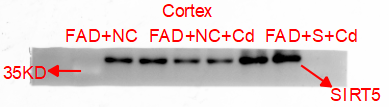


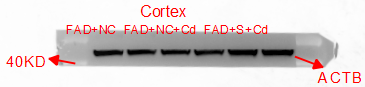


FAD+NC: FAD^4T^+Null FAD+NC+Cd: FAD^4T^+Null+Cd

FAD+S+Cd: FAD^4T^+SIRT5+Cd

Figure S10B


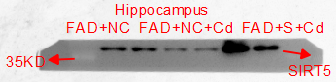


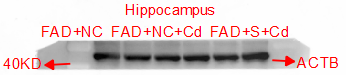


FAD+NC: FAD^4T^+Null FAD+NC+Cd: FAD^4T^+Null+Cd

FAD+S+Cd: FAD^4T^+SIRT5+Cd
